# Constrained reassortment and genotype-specific traits shape the evolutionary landscape of galbut virus

**DOI:** 10.1093/ve/veaf089

**Published:** 2025-11-04

**Authors:** Alexandra H Keene-Snickers, Ali L Brehm, Tillie J Dunham, Taylor A Gelpi, Mark D Stenglein

**Affiliations:** Center for Vector-Borne and Infectious Diseases, Department of Microbiology, Immunology, and Pathology, College of Veterinary Medicine and Biomedical Sciences, Colorado State University, 200 West Lake Street, Fort Collins, CO, 80523, USA; Quantitative Cell and Molecular Biology Graduate Program, Colorado State University, 200 West Lake Street, Fort Collins, CO, 80523, USA; Center for Vector-Borne and Infectious Diseases, Department of Microbiology, Immunology, and Pathology, College of Veterinary Medicine and Biomedical Sciences, Colorado State University, 200 West Lake Street, Fort Collins, CO, 80523, USA; Center for Vector-Borne and Infectious Diseases, Department of Microbiology, Immunology, and Pathology, College of Veterinary Medicine and Biomedical Sciences, Colorado State University, 200 West Lake Street, Fort Collins, CO, 80523, USA; Center for Vector-Borne and Infectious Diseases, Department of Microbiology, Immunology, and Pathology, College of Veterinary Medicine and Biomedical Sciences, Colorado State University, 200 West Lake Street, Fort Collins, CO, 80523, USA; Center for Vector-Borne and Infectious Diseases, Department of Microbiology, Immunology, and Pathology, College of Veterinary Medicine and Biomedical Sciences, Colorado State University, 200 West Lake Street, Fort Collins, CO, 80523, USA

**Keywords:** reassortment, phylogeography, galbut virus, *Drosophila melanogaster*, coinfection, genotype–phenotype, partitivirus

## Abstract

Galbut virus is a remarkably successful virus of the model organism *Drosophila melanogaster*. The ease of capturing galbut virus–infected wild flies presents an opportunity to study persistent virus–host interactions. To better understand galbut virus diversity, evolution, and genotype–phenotype relationships, we screened 957 flies collected from 13 locations across the USA. Galbut virus was detected in every population, and overall, 75% of flies tested positive. We selected 149 flies for shotgun RNA sequencing and recovered 368 coding-complete or near-complete sequences of galbut virus and its likely satellite, chaq virus. Galbut virus sequences clustered phylogenetically into three major clades. Two clades, termed melA and melB, comprised mainly viruses infecting *D. melanogaster*, while the third, simA, included *D. simulans*-infecting viruses. Between-clade pairwise nucleotide identity was as low as 75%, but diversity within clades was low. Geography partly explained phylogenetic clustering: some sequences from the same location were identical or nearly identical, while others were spread throughout trees. Several genotype–phenotype associations emerged, including higher average RNA levels in melA infections and an exclusive association of chaq virus with melA and simA infections. Coinfection was detected in 11% of samples and did not require complete sets of all three galbut virus segments. Coinfection is a prerequisite of reassortment, and there was evidence of reassortment involving all segments and chaq virus. However, reassortment did not occur between clades, despite coinfection, indicating that clades are reproductively isolated. Galbut virus RNA 3 sequences exhibited more involvement in coinfection, greater diversity, and stronger evidence of diversifying selection than the other segments, consistent with a possible role in host modulation. These findings corroborated the ecological success of galbut virus and reveal the need for experiments to uncover the mechanisms underlying reassortment incompatibility and clade-specific phenotypes.

## 1. Introduction

Since the discovery of viruses as filterable agents of disease, virology has largely focused on the harmful impact of viral pathogens (Enquist and Editors of the Journal of Virology 2009, Holmes et al. 2024). This has been warranted; many viruses are terrible pathogens that threaten organism health and economic security. However, emerging evidence from large metagenomic surveys of apparently healthy wild organisms has shown that viral infection is ubiquitous and, in most cases, not associated with overt disease (Webster et al. 2015, Shi et al. 2016, Moustafa et al. 2017, Greninger 2018). A key challenge is to better understand the biological and evolutionary impact of these abundant persistent viruses and the mechanisms that govern their success within hosts and populations.

Over a century of research on the fruit fly *D. melanogaster* has generated wide-ranging biological insight, including into host–pathogen interactions (Letsou and Bohmann 2005, Bellen et al. 2010, Buchon et al. 2014). Insights include discovery of systems to detect and eliminate pathogens and an improved understanding of the ways that hosts and microbes shape each other’s evolution (Weeks et al. 2007, Ramage and Cherry 2015, Douglas 2018, Duxbury et al. 2019, Marques et al. 2024). Studies of *Drosophila* sigmavirus, a vertically transmitted rhabdovirus that establishes lifelong infection, have yielded substantial insight into persistent virus ecology and host–virus interactions (L’Heritier and Teissier 1937, Fleuriet and Periquet 1993, Wayne et al. 1996, Magwire et al. 2011, Longdon and Jiggins 2012).

Metagenomic surveys of wild fly populations have revealed *D. melanogaster* to be, like all organisms, infected by a diverse array of viruses (Webster et al. 2015, 2016, Ortiz-Baez et al. 2021). The most prevalent of these is galbut virus, which is exceptionally common and globally distributed: every wild *D. melanogaster* population examined thus far includes galbut virus–infected flies, and ~60% of individual flies are infected (Webster et al. 2015, Shi et al. 2018, Ortiz-Baez et al. 2021, Wallace and Obbard 2024). The ease of capturing wild *D. melanogaster* and the likelihood that captured flies will be infected by galbut virus provides a promising opportunity to study persistent virus ecology, evolution, and host interactions (Markow and O’Grady 2006).

Galbut virus is a tri-segmented double-stranded (ds) RNA virus related to viruses in the partitivirus family (Ghabrial et al. 2008, Roossinck 2010, Webster et al. 2015, Vainio et al. 2018, Cross et al. 2020). Galbut virus RNA 1 encodes an RNA-dependent RNA polymerase, RNA 2 encodes a capsid protein, and RNA 3 encodes a protein of unknown function. Chaq virus is likely a satellite of galbut virus or may represent an optional fourth galbut virus segment (Webster et al. 2015, Cross et al. 2020). Limited and potentially offsetting fitness impacts have been associated with galbut virus infection, but it’s not clear how these contribute to overall fitness of infected flies in the wild (Cross et al. 2023, Wallace and Obbard 2024).

Galbut virus is vertically transmitted from either infected mothers or infected fathers with high efficiency (Cross et al. 2020). This biparental vertical transmission likely contributes to the galbut virus’s ecological success, and the global distribution of galbut virus may have coincided with *D. melanogaster’s* spread across the world as a human commensal (Fine 1975, Keller 2007, Cross et al. 2020).

Here, we sampled nearly 1000 individual flies across the USA and recovered several hundred galbut and chaq virus sequences. This revealed patterns of diversity, signatures of selection, genotype–phenotype associations, and evidence of coinfection and reassortment.

## 2. Materials and methods

### 2.1. Sample collection

Nine hundred and fifty-seven flies were collected over the summer of 2023 from Maine, Pennsylvania, Ohio, and Colorado, USA. Flies collected in Colorado were trapped in buckets baited with banana and yeast that was covered by organdy mesh (Oriole Textile, fabric #2060). For transportation, the buckets were covered with a lid and moved to the laboratory, where flies were anaesthetised in a −20°C chest freezer. Flies were then collected and stored individually in 96-well plate wells at −20°C until RNA extraction. Flies captured in Maine, Pennsylvania, and Ohio were trapped using 3D-printed fly traps (Keene-Snickers et al. 2025), frozen as pools in 2 ml tubes, and shipped to Colorado. A subset of flies collected in Colorado was isolated from California-grown peaches bought at a grocery store in Fort Collins, CO. Maps were generated using the ggmap R package employing Stamen Terrain map tiles served *via* Stadia Maps (Kahle and Wickham 2013). Map tile data © OpenStreetMap contributors (Open Database License) and © Stamen Design (CC BY 4.0 License).

### 2.2. RNA extraction

RNA was extracted from individual flies using a magnetic bead-based RNA/DNA capture method modified for use on the KingFisher system (ThermoFisher Scientific). Briefly, flies were placed in an assay block (ThermoFisher Scientific, 9761116) with a 2.5 mm ball bearing (McMaster Carr, 1598K22) and 100 μl lysis buffer [20 mM DTT (added fresh), 5 M guanidine thiocyanate, 0.1 M Tris–HCl (pH 7.5), 0.01 M Na_2_EDTA (pH 8)]. Flies were homogenized at 30 Hz for 3 min in a TissueLyszer II (Qiagen, 20.747.0001). The homogenate was moved to a new deep well plate (Thermofisher, 9504050) and combined with a master mix that included: 90 μl SpeedBeads prepared according to manufacturer recommendations (Cytiva, GE65152105050250), 10 μl lysis binding enhancer (Proteinase K 200 μg/ml, 20% glycerol, 0.5% sodium dodecyl sulfate) and 60 μl 100% isopropanol. In separate 96-well standard plates (ThermoFisher, 97002540), 150 μl of wash buffer one [20% ethanol, 900 mM guanidine thiocyanate, 10 mM Tris–HCl (pH 7.5)], 150 μl wash buffer two (200 mM TE, 80% ethanol), and 50 μl of sterile water were added. The extraction was completed on a KingFisher liquid handling robot (Thermo Scientific). Eluted nucleic acids were stored at −80°C until further processing.

### 2.3. RT-qPCR

Complementary DNA (cDNA) was generated using 5 μl RNA, 0.75 μM random pentadecamer primers (IDT), 0.75 μM deoxyribonucleoside triphosphate (dNTP), and water to 14 μl and incubated at 65°C for 5 min. Next, 4 μl 5× buffer (125 mM Tris–HCl pH 8, 125 mM MOPS pH 7.9, 300 mM KCl, 20 mM MgCl_2_, 25% glycerol, and 0.03% NP-40), 1 μl of 100 mM DTT, and 1 μl mashup reverse transcriptase was added and incubated at 50°C for 60 min followed by a heat inactivation step at 80°C for 10 min after which each sample was diluted in 90 μl nuclease-free water. qPCR was conducted using NEB Luna Universal qPCR mix following manufacturer recommendations and cycling conditions (NEB, M3003). Each sample was screened for galbut virus using two primer sets (Supp. Table 1). Galbut virus 1600/1601 primers only detect clade melA sequences. The 2165/2170 primer set detects both clade melA and melB sequences. All wild collected samples were screened using both primers; however, the data presented used data from the 2165/2170 primers. Levels of galbut virus RNA relative to RpL32 mRNA were quantified using the ∆Ct method (Livak and Schmittgen 2001). RpL32 messenger RNA was detected using exon junction-spanning primers (Cao et al. 2016). The Primer BLAST tool predicted that these primers should amplify RpL32 mRNA sequences from *D. melanogaster* and related species (Ye et al. 2012). For instance, there are zero mismatches in the primer binding sites in *D. melanogaster* or *D. simulans* RpL32 mRNA (NM_079843 and XM_016174142) and four mismatches in the *D. innubila* sequence (XM_034628911, two in each primer).

### 2.4. Next-generation sequencing library preparation

One hundred forty galbut virus–positive samples were selected for total RNA sequencing to recover galbut virus sequences. Nine samples collected in Fort Collins, CO, in 2020 and 2021 were also included. Samples were selected to represent diverse locations, galbut virus RNA levels (ranging from low to high), and qPCR melting temperature (ranging from identical to the positive control to 1°C different). Sample concentration was obtained using the Qubit high-sensitivity RNA reagent (ThermoFisher, Q32852), and DNA was removed *via* a DNAse I treatment (NEB B0303S). Prior to library preparation, samples were normalized to 5 ng/μl except where sample concentrations were lower than 5 ng/μl. In these cases, either a different sample was selected, or 5 μl of input RNA was used. Input RNA consisted of total RNA that was neither poly-A selected nor ribosomal RNA depleted. Samples were split into two batches of ≤96 samples and libraries from individual flies were prepared for sequencing using the Kapa RNA Hyper Prep kit following manufacturer recommendations (Roche, 08098093702). Eight cycles of library amplification were performed. Adapters contained unique dual indices (Integrated DNA Technologies xGen UDI/UMI 10005903). HeLa cell total RNA was used as a positive control, and water was used as a negative control for both library preparation, sequencing, and data analysis.

Libraries were pooled evenly by mass, and final library size distribution was determined using an Agilent Tapestation D1000 HS tape. Library pools were sequenced by Azenta on one NovaSeq X 10B lane with 2 × 150 paired-end sequencing.

### 2.5. Identification of galbut virus sequences

We used our lab’s previously described viral metagenomics pipeline (https://github.com/stenglein-lab/taxonomy_pipeline) to identify and validate galbut virus sequences from our NGS datasets (Keene and Stenglein 2024). In brief, adapters and low-quality reads were trimmed using cutadapt 3.5 (Martin 2011). Duplicate read pairs were removed using cd-hit-dup v4.8.1 using parameter -e 2 (Fu et al. 2012). Fastqc 0.11.9 (Andrews et al. 2012) was used to assess read quality. Reads mapping to the *D. melanogaster* genome or transcriptome (GCF_000001215.4) (Hoskins et al. 2015) were removed with bowtie2 2.4.5 (Langmead and Salzberg 2012), using parameters --local, --sensitive, and a minimum alignment score of 40. The number of host-mapping reads was tabulated using the ‘reads mapped’ output of the samtools stats command run with the host-mapping bam files as input (Li et al. 2009). The number of reads mapping to the RpL32 mRNA (NM_079843.4) was extracted from the output of samtools coverage with the same bam files as input. Remaining non-host-mapping reads were assembled using spades 3.15.4 using default parameters (Prjibelski et al. 2020). Candidate galbut virus contigs were identified by querying the National Center for Biotechnology Information (NCBI) nucleotide database using BLASTn 2.12.0 (Altschul et al. 1997). Draft galbut virus sequences were manually validated by remapping of trimmed reads using bwa mem aligner version 0.7.17 (Li and Durbin 2010). Final validated galbut virus sequences were submitted to the NCBI nucleotide database and raw NGS data to the NCBI sequence read archive repository (see Data Availability statement below). We identified coinfecting sequences as coding-complete sequences that were sufficiently dissimilar to the other sequences from the same sample that they attracted distinct sets of reads during competitive mapping.

### 2.6. Molecular species identification

To confirm the morphology-based species assignment of sequenced samples, we used our lab’s species identification pipeline (https://github.com/stenglein-lab/species_id) (Keene and Stenglein 2024). This pipeline maps trimmed reads, described above, to a set of 529 cytochrome c oxidase subunit 1 (c) sequences from across the Drosophilidae family. We collected this set of sequences on 13 August 2024 as follows: We used the CO1 coding sequence from NC_024511 as a megabast query to the NCBI core_nt nucleotide database. We restricted megablast results to sequences from Drosophilidae and to alignments with >90% query coverage. We used cd-hit-est to collapse sequences that shared >99% pairwise nucleotide identity to create a set of representative CO1 sequences, from which we created a bowtie2 index. We mapped trimmed reads to this index with bowtie2 2.4.5 using parameters --end-to-end, --no-discordant, --no-mixed, and --maxins 600 and used samtools v1.16 to remove reads with a mapping quality lower than 20. The number of reads mapping to each CO1 sequence and the average percent identify of mapped reads was tabulated.

### 2.7. Maximum likelihood trees

We built trees from sequences generated here and all available Genbank coding-complete galbut virus and chaq virus sequences from individual flies with a known location and date of collection. Alignments were generated using MAFFTT v7.490 with default parameters (Katoh and Standley 2013). IQ-TREE v2.3.6 was used to generate maximum likelihood trees (Nguyen et al. 2015). IQ-TREE parameters were: -B 1000 and -alrt 1 000. Trees were midpoint rooted using the phytools R package and then visualized using the interactive Tree of Life (iTol) tool (Letunic and Bork 2024, Revell 2024).

### 2.8. Recombinant detection analysis

We used the Recombination Detection Program v4.101 (Martin and Rybicki 2000, Martin et al. 2021) to evaluate alignments for evidence of recombination prior to tree construction. This tool identified one candidate recombination event in the chaq virus alignment and another in the galbut virus RNA 3 alignment. We visualized pairwise percent identity plots between the candidate recombinant sequences and the putative parental sequences. In both cases, manual inspection did not support the conclusion that sequences corresponded to legitimate recombinant genotypes.

### 2.9. Selection analysis

We used the Fixed Effects Likelihood (FEL) method described by Kosakovsky Pond and Frost to identify sites with evidence of diversifying or purifying selection (Kosakovsky Pond and Frost 2005). Protein alignments from each galbut virus segment and chaq virus were input to the Datamonkey webserver using default parameters and all branches selected (Weaver et al. 2018, Kosakovsky Pond et al. 2020). We used a *P*-value of <0.1 for evidence of selection as a relaxed constraint that has been shown to have reasonable power to detect true positives while maintaining a low false-positive rate (Kosakovsky Pond and Frost 2005).

### 2.10. Detection of reassortment

Tanglegrams were generated in R/RStudio using the phytools and ape packages (Paradis and Schliep 2018, Revell 2024). Branches shorter than 0.001 were converted to polytomies to avoid false-positive signals of reassortment from short branches. Reassortment was confirmed using the GARD tool on the Datamonkey webserver (Kosakovsky Pond et al. 2006, Weaver et al. 2018). This tool is designed to detect evidence of recombination given a set of aligned sequences but can be used to detect reassortment given concatenated input sequences. We concatenated sequences for each pair of galbut virus and chaq virus segments from singly infected samples and used the cd-hit-est tool to collapse concatenated sequences that shared over 99% pairwise identity. We aligned these concatenated sequences using the MAFFT aligner using default settings and input alignments to the GARD tool using default parameters and normal run mode. For all segment pairs, GARD identified a single breakpoint at the junction of the two concatenated segments. This did not identify specific instances of reassortment but confirmed that reassortment occurred between all pairs of galbut virus and chaq virus segments.

### 2.11. Statistical tests

Differences in galbut virus prevalence between locations and across timepoints were evaluated using pairwise Fisher’s exact tests with *P*-values adjusted for multiple testing using the Holm–Bonferroni method. We evaluated differences in plus and minus strand coverage levels for different galbut virus segments and chaq virus, using pairwise Wilcoxon rank-sum tests with *P*-values adjusted for multiple testing using the Holm–Bonferroni method. To evaluate within-segment nucleotide diversity, we calculated pairwise nucleotide identity values for each pair of sequences for each galbut virus RNA and for chaq virus. We compared the resulting distributions of pairwise nucleotide identities using bootstrap testing as implemented in the boot.t.test method in the MKinfer R package (Kohl 2024).

### 2.12. Analysis of geographic population structure

We used the nearest-neighbour statistic, *S*_nn_, to evaluate the extent to which geography shaped galbut virus population structure (Hudson 2000). *S*_nn_ measures how often the nearest neighbours of a sequence are from the same geographic location. The nearest neighbours of a sequence are those other sequences that are separated by the smallest genetic distance. We calculated *S*_nn_ in R using the algorithm described by Hudson with distances calculated using the TN93 model and the multiple sequence alignments described above (Tamura and Nei 1993, Hudson 2000). We assigned geographic labels based on the US state or country for non-US locations. We calculated *P*-values using permutation tests (5000 permutations) with location labels randomized without replacement.

**Figure 1:**
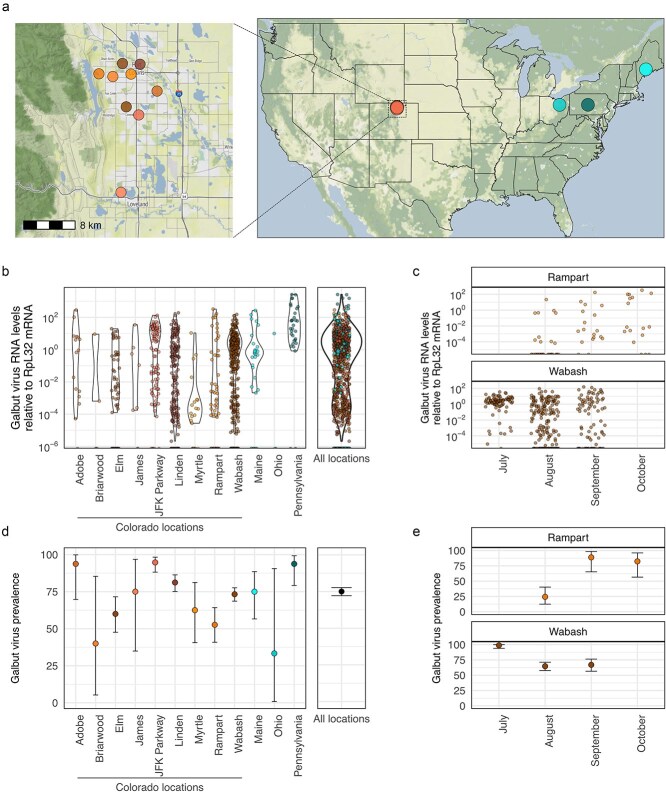
Galbut virus prevalence is high overall and varies across location and time. (a) Locations in the United States where wild *Drosophila* were collected in 2023. Left map shows locations in and around Fort Collins, CO. (b) Galbut virus RNA levels normalized to RpL32 mRNA levels in individual flies from Colorado and other states. Negative samples are plotted at the minimum *Y*-axis value. Colorado locations are labelled using street names. Combined values from all samples are shown in the right panel. (c) Galbut virus RNA levels in individual flies over time at the Rampart and Wabash locations. (d) Galbut virus prevalence at each location expressed as percent of RpL32-positive flies that were positive for galbut virus by RT-qPCR. Overall prevalence in all samples is shown on the right. Exact binomial confidence intervals are shown. (e) Galbut virus prevalence over time at the rampart and Wabash locations. Exact binomial confidence intervals are shown. Base map © OpenStreetMap contributors (ODbL), map tiles by Stamen Design (CC BY 4.0), served *via* Stadia Maps.

## 3. Results

### 3.1. Galbut virus prevalence varies by location and time

To better understand galbut virus prevalence and diversity, we sampled flies from nine locations in Larimer County, CO, and from one location each in Maine, Pennsylvania, and Ohio (Fig. 1a). We used buckets baited with banana and yeast to collect flies in Colorado, and volunteers used 3D-printed fly traps to collect flies from Maine, Ohio, and Pennsylvania (Keene-Snickers et al. 2025). Overall, we collected and tested 957 individual flies from all locations during the summer of 2023. Sampled flies were morphologically consistent with being *D. melanogaster*.

We screened RNA from individual flies for galbut virus *via* RT-qPCR (Fig. 1). Ribosomal protein L32 (RpL32) mRNA levels were used to normalize galbut virus RNA levels and served as a positive control. Of the 957 flies tested, 952 were positive for RpL32 mRNA, indicating successful RNA extraction and sufficient relatedness to *D. melanogaster* for our RpL32-targeting primers to work. Overall, 714 flies (75%) were positive for galbut virus RNA by RT-qPCR (Fig. 1b). We observed a bimodal distribution of galbut virus levels in infected flies, with levels in individual flies spanning several orders of magnitude relative to levels of RpL32 mRNA (Fig. 1b). The higher mode corresponded to 4.1 galbut virus RNA copies per RpL32 mRNA and the lower mode to 1 galbut virus RNA per 3907 RpL32 mRNA copies.

At Colorado locations, galbut virus prevalence varied from 40% to 100% (Fig. 1d). In Maine, Ohio, and Pennsylvania, prevalence was 77%, 33% and 97%, respectively (Fig. 1d). The prevalence differed significantly between some locations (Supp. Table 2). For instance, the prevalence at the JFK Parkway location (94.7%, binomial confidence interval [88.1%–98.3%]) was significantly higher than overall prevalence at the Rampart location (52.6% [40.8%–64.2%]; Fisher’s exact *P* = 5.3 × 10^−9^).

We repeatedly sampled flies from the Wabash and Rampart Colorado locations over three months (Fig. 1c and e). A Fisher’s exact test found significant differences in galbut virus prevalence at these locations over time (Supp. Table 3). Prevalence rose from 24% in August to 89% in September at the Rampart location (Fisher’s exact *P* = 1.8 × 10^−5^) and then decreased to 82% in October (*P* = 1 relative to September). Similarly, at the Wabash location, there was a significant decrease in prevalence between the first 2 months of sampling, July and August (*P* = 3.9 × 10^−12^), but not between August and September (*P* = 1). Thus, galbut virus prevalence varied significantly between locations and over time. However, what caused these fluctuations was not clear and, in one location, prevalence rose over time, while prevalence at the other location fell.

### 3.2. Recovery of coding complete galbut and chaq virus and genome sequences from 101 infected flies

We selected 140 of the 957 samples for recovery of galbut virus genome sequences using total RNA sequencing. Samples were selected to provide a broad representation of geography, RNA level, and galbut virus genotype, with genotype inferred from the pattern of qPCR positivity from our two galbut virus–targeting primer sets and qPCR melting curve differences (Supp. Table 4). We also sequenced nine flies that had been collected in 2020 and 2021 from Colorado to expand the temporal range, for a total of 149 individual flies.

We mapped quality- and adapter-trimmed reads to a set of representative sequences to recover galbut virus and chaq virus genome sequences. Thirty-one samples produced partial galbut virus sequences, and 17 had fewer than 10 galbut virus mapping reads. This was not unexpected, as we had selected flies with a range of RNA levels for sequencing. From the 101 samples with sufficient galbut virus–mapping reads, we recovered 368 coding-complete or near-coding-complete sequences (Supp. Fig. 1). Sequences were supported by ample coverage, with a median coverage depth across all segments of 728× (range: 5×–204 364×; Supp. Fig. 1).

To confirm the species identity of sequenced flies, we competitively mapped reads from each dataset to a set of cytochrome c oxidase subunit 1 (CO1) sequences representing species from across the Drosophilidae family. All samples were identified as *D. melanogaster* except for one sample from Maine, ME-M-3, which was identified as *D. simulans* (Supp. Table 5).

Galbut virus RNA 1 levels measured by RT-qPCR correlated well with the number of galbut virus RNA 1-mapping reads per RpL32-mapping read (Supp. Fig. 2). Linear regression of log10-transformed relative RNA levels yielded a slope of 1.1 and an intercept of −1.18 (*R*^2^ = 0.83; Supp. Fig. 2a). The number of galbut-virus RNA 1 mapping reads per million host-mapping reads (RPM) in each dataset was slightly less well correlated, with a coefficient of determination of 0.72 (Supp. Fig. 2b). The negative intercepts indicated that RT-qPCR under-quantified galbut virus RNA levels relative to NGS. Nevertheless, these correlations demonstrated overall concordance between these independent measures of galbut virus abundance and confirmed that relative galbut virus RNA levels spanned several orders of magnitude in individual flies.

### 3.3. Galbut virus diversity and evolutionary history

**Figure 2:**
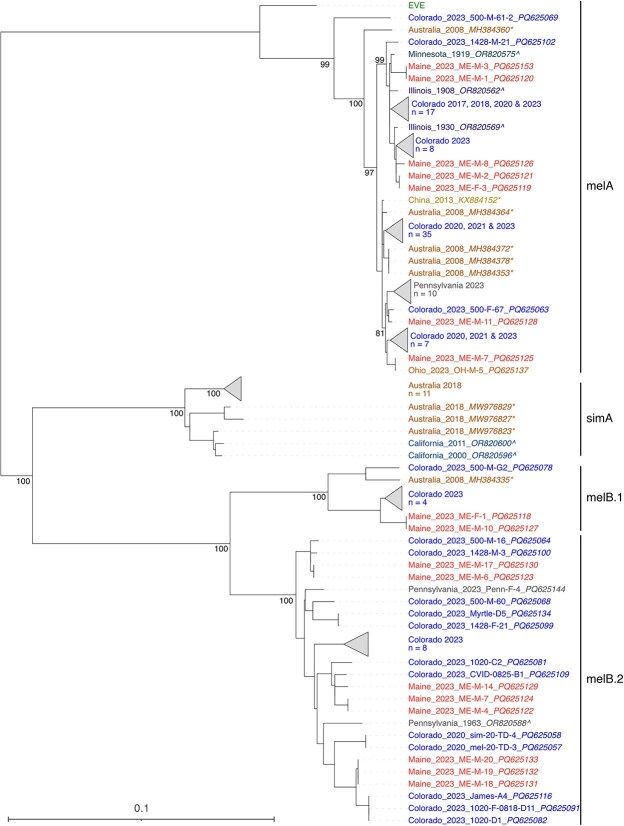
Midpoint-rooted maximum likelihood phylogenetic tree of galbut virus RNA 1 coding-complete nucleotide sequences. Sample names are coloured by location. Sequences generated by others are indicated with an asterisk (^*^). Sequences from museum specimens are indicated by a caret (^) (Keene and Stenglein 2024). Endogenized viral element (EVE) indicates an endogenized galbut virus sequence present in some *D. melanogaster* genomes (Wallace et al. 2021). Support values for select nodes are indicated. Accession numbers are noted except where groups of closely related sequences were collapsed.

**Figure 3:**
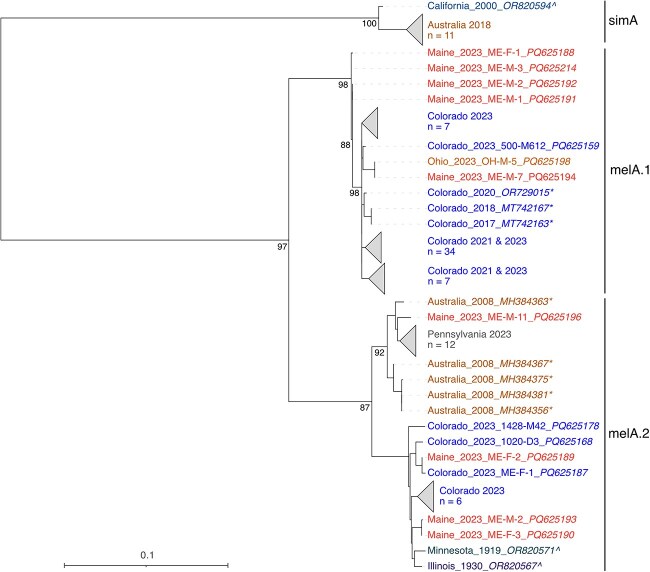
Midpoint rooted maximum likelihood phylogenetic tree of coding-complete chaq virus nucleotide sequences. Sample names are coloured by location. Sequences generated by other investigators are indicated with an (^*^). Sequences from museum specimens are indicated by a (^) (Keene and Stenglein 2024). Support values for select nodes are indicated. Accession numbers are noted except where groups of closely related sequences were collapsed.

We created maximum likelihood phylogenetic trees using the galbut virus sequences generated here and those available in the NCBI nucleotide database (Figs 2 and 3 & Supp. Figs 3–6). We included coding complete sequences that derived from a single fly (not a pool), and had a known collection year and location. Existing galbut virus sequences derived from *D. melanogaster* and *D. simulans* from the USA, Australia, and China and included sequences from museum specimens (Shi et al. 2016, Shi et al. 2018, Ortiz-Baez et al. 2021, Keene and Stenglein 2024). We also generated galbut virus RNA 2 and 3 sequences from Sequence Read Archive (SRA) datasets from Australian *D. simulans* for which galbut virus RNA 1 and Chaq virus sequences were previously available (Ortiz-Baez et al. 2021). These new Australian RNA 2 and RNA 3 sequences have been deposited to the NCBI nucleotide database under accessions PV185866-PV185894.

Galbut virus sequences clustered into several major genotypes. We defined genotypes as groups of sequences that shared greater than ~95% pairwise nucleotide identity and that formed monophyletic clades. Galbut virus RNA 1 and RNA 2 sequences grouped into three major genotypes, two containing viruses from *D. melanogaster* and the third containing viruses from *D. simulans*, which we termed melA, melB, and simA (Fig. 2 & Supp. Figs 3 and 4) (Keene and Stenglein 2024). The sole exception to this was sample ME-M-3, which was a *D. simulans* with melA galbut virus sequences (Fig. 2). For RNA 1 and RNA 2, the clade B sequences were further subdivided into clades that we named melB.1 and melB.2 (Supp. Figs 3 and 4). RNA 3 also had the three major genotypes, including subclades melB.1 and melB.2. Additionally, RNA 3 melA was subdivided into two clades, melA.1 and melA.2 (Supp. Fig. 5). Chaq virus clustered into three clades, which we termed melA.1, melA.2, and simA (Fig. 3 & Supp. Fig. 6).

Geography partly explained phylogenetic clustering (Figs. 2 and 3 & Supp. Figs 3–6). Subsets of samples from the same location and the same date tended to form clusters of identical or nearly identical sequences. For example, most RNA 1 sequences from flies collected from Pennsylvania formed a monophyletic cluster (Fig. 2). Groups of nearly identical sequences from Colorado similarly clustered together. We used the nearest neighbour statistic, *S*_nn_, to evaluate geographic population structure (Hudson 2000). *S*_nn_ provides an estimate of how often the nearest genetic neighbours of a sequence come from the same location, here defined as the same US state or other country. The *S*_nn_ for galbut virus RNA 1, RNA 2, RNA 3, and chaq virus were 0.85, 0.87, 0.87, and 0.91, respectively (all *P* < 2 × 10^−4^ by permutation testing). These values are consistent with a high degree of geographical population structure: an *S*_nn_ of 1 occurs when nearest neighbours always come from the same location. At the same time, sequences from individual locations were spread throughout the trees (Figs 2 and 3 & Supp. Figs 3–6). These patterns were consistent with both location-specific lineages that presumably reflected recent shared ancestry through vertical transmission and widespread distribution of different galbut virus genotypes (Cross et al. 2020). Sequences from museum specimens were similar to but not identical to contemporary sequences (Figs 2 and 3 & Supp. Figs 3–6) (Keene and Stenglein 2024).

### 3.4. Galbut virus coinfection is common and does not require complete sets of all three segments

**Figure 4:**
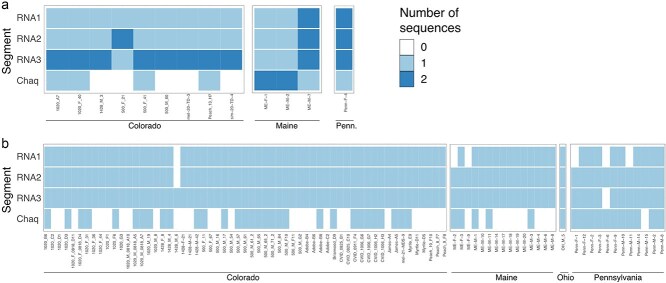
Galbut virus coinfection is common and does not require complete sets of all three segments. Heatmaps depicting the number of coding-complete sequences, all of which were deposited to Genbank, recovered from (a) samples with evidence of coinfection or (b) samples with no evidence of coinfection. Many samples with high galbut virus coverage did not contain chaq virus sequences.

Eleven percent (13/118) of samples that produced coding complete galbut virus sequences exhibited evidence of coinfection (Fig. 4 & Supp. Figs 1 and 3–6). This underestimated the true coinfection rate as it only considered coding-complete sequences, and there was evidence of lower coverage, partial sequences in many datasets. Coinfection involved various numbers of genotypically distinct galbut virus and chaq virus segments (Fig. 4). Two samples, ME-M-7 and Penn-F-4, corresponded to simple coinfections, with two complete sets of all three galbut virus segments. Sample ME-M-7 produced six galbut virus sequences: a melA sequence and a melB sequence for each of the three galbut virus RNAs (Fig. 4, Supp. Figs 3–6). These were the only samples that produced multiple coding-complete RNA 1 genotypes. Other coinfections yielded variable numbers of coding-complete sequences for the different segments. For instance, sample 500-F-41 yielded one coding-complete sequence for RNA 1, RNA 2, and chaq virus but two coding-complete RNA 3 sequences, which shared 87.5% pairwise nucleotide identity (Fig. 4; Supp. Fig. 5). RNA 3 was the segment most often present as a coinfection.

Some samples that produced coding-complete RNA 2 and RNA 3 sequences only produced partial RNA 1 sequences (Fig. 4). We had prepared libraries using a strand-specific protocol, so we could assess levels of plus (+) and minus (−) strand mapping reads separately (Supp. Fig. 7). Galbut virus genomic RNA is presumed to be double-stranded, so levels of -strand RNA can be used as a proxy for genomic RNA levels, whereas +strand RNA reflects levels of genomic RNA and mRNA, and nearly all galbut virus RNA is +strand (Supp. Fig. 7a). Average levels of RNA 1 −strand were not significantly lower than −strand levels of other segments (Supp. Fig. 7c). However, RNA 1 exhibited the lowest ratio of +strand RNA copies per −strand RNA (Wilcoxon rank-sum adjusted *P* < 10^−6^ compared to all other segments; Supp. Fig. 7d). This was consistent with less efficient transcription of RNA 1 or a shorter half-life of RNA 1 transcripts, which likely contributed to recovery of fewer coding complete RNA 1 sequences.

Some datasets, including datasets with plenty of galbut virus-mapping reads, did not produce chaq virus sequences (Fig. 6). This corroborated prior reports that chaq virus is not present in every galbut virus infection and reflects chaq virus’s status as a likely satellite of galbut virus (Webster et al. 2015, Shi et al. 2018, Cross et al. 2020, Ortiz-Baez et al. 2021).

### 3.5. Galbut virus RNA 3 is more diverse than RNA 1 or 2 and exhibits more evidence of diversifying selection

Galbut virus segments exhibited varying levels of pairwise sequence divergence, with all segments exhibiting significantly different distributions (all *P* < 2 × 10^−4^ by bootstrap testing; Supp. Fig. 8). RNA 3 sequences exhibited the most diversity and RNA 2 the least (Supp. Fig. 8). The median pairwise percent identity between RNA 1, RNA 2, RNA 3, and chaq sequences was 89.7%, 91.7%, 83.0%, and 91.1%, respectively (Supp. Fig. 8). Because there were no clade B chaq virus sequences (see below), the diversity of chaq virus sequences was underestimated by this measure relative to galbut virus sequences.

**Figure 5:**
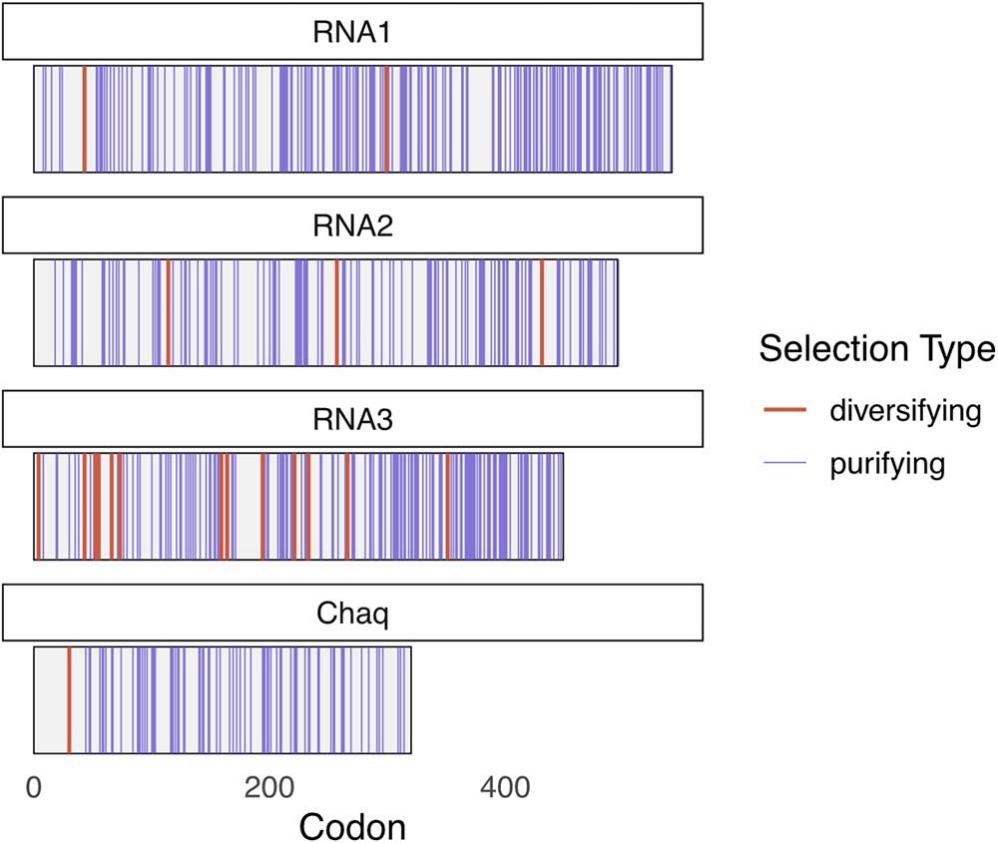
Galbut virus RNA 3 sequences exhibited more evidence of diversifying selection, but overall galbut and chaq virus sequences exhibit a preponderance of purifying selection. Location of diversifying and purifying selection is shown for each galbut virus segment and chaq virus.

We examined galbut and chaq virus sequences for evidence of natural selection using the FEL model, which estimates synonymous and nonsynonymous substitution rates for each site in an alignment and tests whether these rates differ significantly (Kosakovsky Pond and Frost 2005). Botella et al. reported purifying selection in the RNA 1 and RNA 2 segments of a fungus-infecting partitivirus (Botella et al. 2015). Consistent with this, we found evidence of purifying selection in all segments, with 187, 126, 154, and 76 sites experiencing purifying selection in galbut virus RNA 1, 2, 3, and chaq virus, respectively (Fig. 5 & Supp. Table 6). There was little evidence of diversifying selection in galbut virus RNA 1, RNA 2, or chaq virus with 2, 3, and 1 diversifying sites identified, respectively (Fig. 5 & Supp. Table 7). In contrast, galbut virus RNA 3 showed more evidence of diversifying selection, with 13 sites identified (Fig. 5 & Supp. Table 7).

### 3.6. Galbut virus genotype melA replicates to higher levels than genotype melB regardless of chaq virus infection

**Figure 6:**
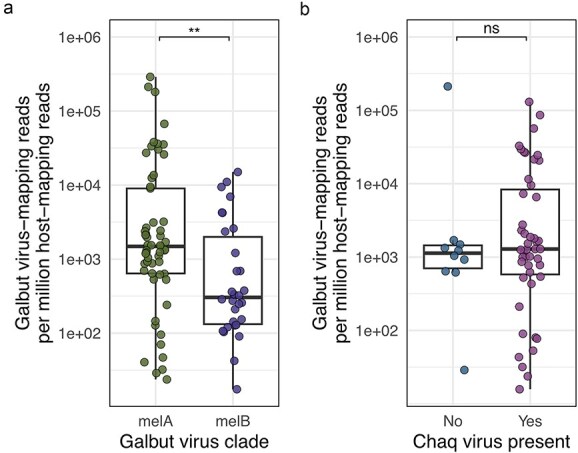
Galbut virus genotype melA replicates to higher levels than genotype melB. (a) Boxplot and scatter plots of total galbut virus reads per million host-mapping reads for clades melA and melB from singly infected *D. melanogaster* samples. Each point represents the value from a single fly. (b) Total galbut virus mapping reads per million host-mapping reads in *D. melanogaster* clade melA infections with and without chaq virus. Significance levels of Wilcoxon rank-sum test p-values are indicated: ^**^: *P* < .01, ns, not significant: *P* > .05.

Previous RT-qPCR-based observations from our lab suggested that galbut virus genotype melA replicated to higher levels than genotype melB. We corroborated this observation using galbut virus−mapping read counts normalized to total read counts (galbut virus mapping reads per million host-mapping reads; RPM). To remove the potentially confounding effect of coinfection, we focused this analysis on singly infected samples (Fig. 4). Galbut virus genotype melA median RPM values were 4.9× higher than in genotype melB infections (Fig. 6a, Wilcoxon *P*-value = 5.0 × 10^−3^). Despite this higher median level, some galbut clade melA samples had very low RNA levels, below 100 RPM. We next evaluated whether the presence of chaq virus increased levels of galbut virus RNA. There was no difference in median galbut virus RPM levels between infections with or without chaq virus (Fig. 6b; Wilcoxon *P*-value = .60).

### 3.7. Galbut virus reassortment occurs within but not between genotypes

Reassortment—the shuffling of genome segments following coinfection is common for viruses with segmented genomes (Lowen 2018). The short branches in galbut virus trees and high frequency of coinfection complicated detection of reassortment. We therefore excluded samples with evidence of coinfection and collapsed very short branches in trees into polytomies to mitigate these potential confounders (Barrat-Charlaix et al. 2022).

**Figure 7:**
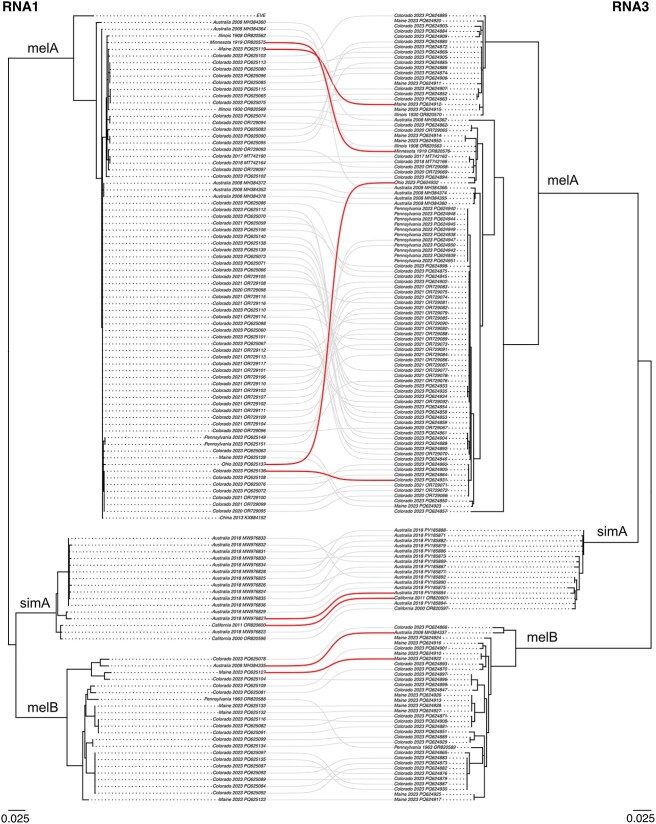
Galbut virus reassortment involving RNA 1 and RNA 3. Maximum likelihood midpoint rooted trees showing all RNA 1 and RNA 3 nucleotide sequences from singly infected samples. Branches with lengths <0.001 were converted to polytomies. Sequences from the same sample are connected by lines. Selected examples of phylogenetic discordance of samples consistent with of pairs reassortment are highlighted.

**Figure 8:**
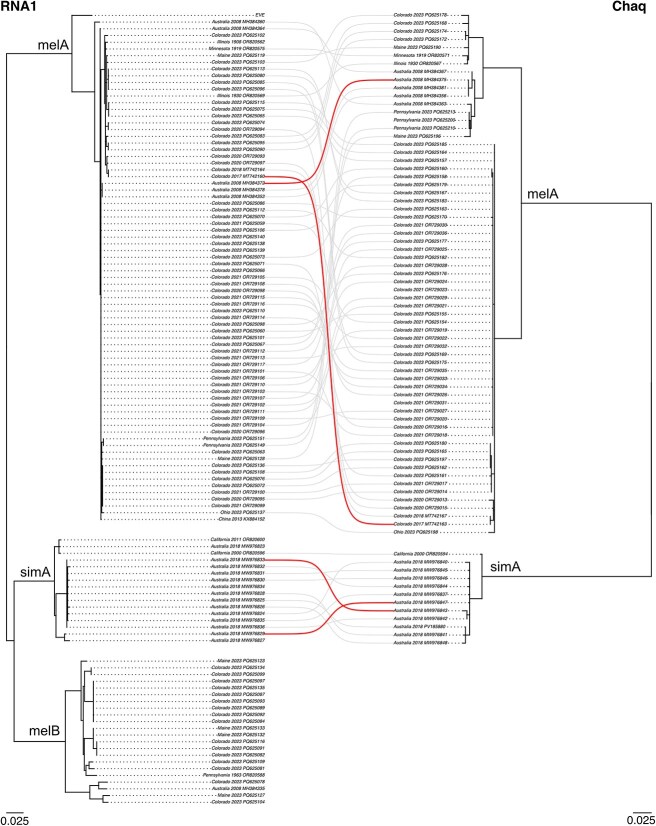
Reassortment involving galbut virus RNA 1 and chaq virus. Maximum likelihood midpoint rooted trees showing all RNA 1 and chaq virus nucleotide sequences from samples without evidence of coinfection. Branches with lengths <0.001 were converted to polytomies. Sequences from the same sample are connected by lines. Selected examples of phylogenetic discordance of pairs of samples consistent with reassortment, or a reassortment-like process, are highlighted.

There were many examples of phylogenetic discordance in segment cophylogenies that were consistent with galbut virus reassortment (Figs 7 and 8 & Supp. Figs 9–12). For instance, RNA 1 sequences PQ625119 and OR820575 shared 99.5% pairwise nucleotide identity. However, the RNA 3 virus sequences from these flies (PQ624912 and OR820576) only shared 88.9% identity (Fig. 7). Similarly, RNA 1 sequences MT742160 and MH384372 shared 98.7% pairwise identity while the corresponding chaq virus sequences (MT742163 and MH384375) only shared 91.9% identity (Fig. 8). These sequences were phylogenetically positioned in different, well-supported clades in the corresponding trees.

Reassortment occurred within all the major galbut virus clades and involved all combinations of galbut virus segments and chaq virus. However, there was no evidence of reassortment between genotypes (Figs 7 and 8 & Supp. Figs 9–12). This included no evidence of reassortment between galbut viruses from the major *D. melanogaster* and *D. simulans* clades and no evidence of reassortment between *D. melanogaster* melA and melB clades. Coinfection by melA and melB viruses occurred (Supp. Figs 3–6), indicating that there was an opportunity for reassortment to have occurred, were it possible.

### 3.8. Chaq virus is associated only with galbut virus clade melA and simA infection

Chaq virus was exclusively associated with galbut virus melA and simA infections (Fig. 8 & Supp. Figs 11 and 12). There were two instances of flies infected by clade B galbut virus that yielded chaq virus sequences, but these two instances were samples with complete galbut virus coinfections of genotypes melA and melB (ME-M-7 & Penn-F-4). Given the coinfection status of all these samples, we concluded that the chaq virus was only associated with genotype melA and simA infections. This limited association was evident in cophylogenies of singly infected samples (Fig. 8 & Supp. Figs 11 and 12).

## 4. Discussion

In this study, we screened nearly 1000 individual flies for galbut virus, a ubiquitous persistent virus of *D. melanogaster*, and generated several hundred new galbut and chaq virus sequences (Webster et al. 2015). Galbut virus prevalence varied from 33% to 100% across locations, but on average, 75% of flies were infected, underscoring the remarkable success of this virus (Fig. 1). Prevalence varied significantly as a function of time and location but no consistent patterns to explain the fluctuating prevalence were evident (Fig. 1). Longitudinal sampling of more locations could shed light on the factors that determine galbut virus prevalence over space and time. Some of the flies we sampled may have been *D. simulans* instead of *D. melanogaster*, which are difficult to distinguish morphologically (Sturtevant 1919). *Drosophila melanogaster* and *simulans* have been reported to be infected by galbut virus at similar high frequencies, and the prevalences reported here may represent combined estimates (Webster et al. 2015).

Galbut virus RNA levels within individual flies exhibited a bimodal distribution that spanned a wide range of relative abundance (Fig. 1). Most infected flies had high viral RNA levels, on average approximately four times higher than levels of RpL32 mRNA, a very highly expressed transcript (Fig. 1b) (Brown et al. 2014). Another subset of infected flies had much lower galbut virus RNA levels, with only about one galbut virus RNA per 4000 RpL32 mRNAs (Fig. 1b). Some of the difference in galbut virus RNA levels was attributable to lower replication of melB genotype viruses (Fig. 8b). The extent to which other factors, including genetic host resistance, influence galbut virus replication levels remains to be determined (Palmer et al. 2018).

The similarity of sequences within clades may indicate that the galbut virus is well adapted to its host (Figs 2 and 3 and Supp. Figs 3–6) (Simmonds et al. 2019). The fact that sequences from museum specimens from over a hundred years ago were nearly identical to contemporary sequences supports this observation, as does the preponderance of purifying selection (Fig. 5 & Supp. Figs 3–6) (Keene and Stenglein 2024).

The most diverse galbut virus segment was RNA 3, which exhibited more nucleotide diversity than RNA 1 or RNA 2 and more evidence of diversifying selection (Fig. 5 & Supp. Fig. 8). The protein encoded on RNA 3 has no known function and no detectable sequence or structural similarity to proteins with known function. It may be that RNA 3 is involved in a host antagonistic process such as immune modulation (Daugherty and Malik 2012, Goic and Saleh 2012, Swevers et al. 2013).

We interpreted the lack of reassortment between viruses from the three major galbut virus clades as evidence of reproductive isolation, i.e. lack of gene flow between viruses from these groups (Figs 7 and 8 & Supp. Figs 9–12) (McDonald et al. 2016, Lowen 2018). The separation between *D. melanogaster* and *D. simulans* viruses may reflect a reliance on vertical transmission and the reproductive isolation of the hosts (Barbash 2010). Alternatively, if the galbut virus can be horizontally transmitted, then host-specific barriers to replication may exist (Wallace and Obbard 2024). A single fly (ME-M-3) identified by CO1 genotype as *D. simulans* was infected by a melA virus. The significance of this observation could be clarified through the recovery of galbut virus sequences from more *D. simulans*.

We sampled flies that were coinfected by viruses from the melA and the melB clades, which would provide an opportunity for reassortment to occur, were it possible. Some coinfections combined a single RNA 1 and a single RNA 2 with RNA 3s from both *melanogaster* clades. For instance, the infection in fly Penn-M-2 consisted of a melA RNA 1 and RNA 2 and both a melA and a melB RNA 3 (Fig. 4, Supp. Figs 3–5). Similarly, fly 500-M-60 was infected by a melB RNA 1 and RNA 2 and both a melA and a melB RNA 3 (Fig. 4, Supp. Figs 3–5). These examples suggested that the RNA-dependent RNA polymerase encoded by RNA 1 is capable of replicating RNA 3 s from both clades and the capsid encoded by RNA 2 is capable of encapsidating both RNA 3 types. So, a failure of these basic steps of the viral lifecycle does not seem to underlie the inability of melA and melB viruses to reassort.

Several genotype–phenotype associations emerged from this study. First, we found that the chaq virus was strictly associated with melA and simA galbut virus infections (Fig. 8, Supp. Figs 11 and 12). This presumably reflects the loss of chaq virus during the evolution of the melB galbut virus lineage. MelB-encoded polymerase and capsid proteins must have lost the ability to replicate the melA chaq RNA; otherwise, reassortment could have allowed the melA chaq virus to associate with the melB galbut virus (Chen et al. 2009). Second, clade melA RNA levels were, on average, significantly higher than melB RNA levels (Fig. 6a). A possible explanation for this could be that the presence of chaq virus increases replication of melA galbut virus, but we did not observe such an effect (Fig. 6b) (Parks et al. 1968, Qiu and Karen-Beth 2001). Additional work will be needed to define the molecular mechanisms underlying these genotype-associated phenotypic differences.

We found evidence of galbut virus coinfection in 11% of flies (Fig. 4). Only two of these cases corresponded to simple coinfection by two viruses (two sets of three segments). Instead, coinfections were characterized by the presence of one or more extra segments. This type of coinfection involving variable numbers of different segments has been observed for other segmented RNA viruses (Hepojoki et al. 2015, Stenglein et al. 2015, Kapuscinski et al. 2021, Albrecht et al. 2022, Cuypers et al. 2022). RNA 3 was the most common segment to be present as more than one distinct genotype. RNA 2 and chaq virus were the next most common coinfecting segment. Multiple RNA 1 segments were only found in complete coinfections (Fig. 4). It is possible that maintaining multiple RNA 3 genotypes in a single infection confers some kind of advantage. Alternatively, molecular details of how the different RNA segments are replicated or transmitted may contribute to their differential abundances and persistence. The low diversity of RNA 1 and 2 made it more difficult to identify coinfecting genotypes for these segments, and the lower levels of RNA 1 plus strand RNA likely contributed to less efficient recovery of coding-complete RNA 1 sequences. Whether the presence of multiple genotypes of one segment changes infection outcomes remains to be determined.

Persistent viral infections are increasingly recognized as important modulators of host biology and as the cause of diseases that manifest over the long term (Goic and Saleh 2012, McGlynn et al. 2021, Bjornevik et al. 2022, Wallace and Obbard 2024). *Drosophila melanogaster* in nature are more likely than not to be infected by galbut virus. Despite this, and despite over a century of *Drosophila* research, much remains unknown about how galbut virus infection shapes the biology and evolution of its host. This study contributes to a stronger understanding of the ecology and evolutionary history of this widespread infection of an important model organism (Markow 2015).

## Supplementary Material

Supplemental_Figure_1_coverage_plots_veaf089

Supplemental_Figure_2_qPCR_vs_NGS_quantification_veaf089

Supplemental_figure_3_full_RNA1_tree_veaf089

Supplemental_figure_4_full_RNA2_tree_veaf089

Supplemental_figure_5_full_RNA3_tree_veaf089

Supplemental_figure_6_full_chaq_tree_veaf089

Supplemental_figure_7_segment_strand_levels_veaf089

Supplemental_figure_8_pairwise_pct_identity_by_segment_veaf089

Supplemental_figure_9_RNA1_RNA2_tanglegram_veaf089

Supplemental_figure_10_RNA2_RNA3_tanglegram_veaf089

Supplemental_figure_11_RNA2_Chaq_tanglegram_veaf089

Supplemental_figure_12_RNA3_Chaq_tanglegram_veaf089

Supplemental_Tables_1_to_7_veaf089

Supplemental_Figure_Legends_veaf089

## Data Availability

Sequencing data have been deposited in the National Center for Biotechnology and Information Sequence Read Archive (SRA) database under accessions SRR30712177-SRR30712331 and in the Nucleotide database under accessions PQ624841-PQ625194, PQ625196-PQ625214, and PV185866-PV185894. The scripts used to analyse the data, raw RT-qPCR data statistical analyses, and metadata can be found at this GitHub repository: https://github.com/LKeene/DiverseCollections.
