# Supplementary figures and images for: Constrained reassortment and genotype-specific traits shape the evolutionary landscape of galbut virus
Source: Virus Evol. 2025 Nov 4;11(1):veaf089. doi: 10.1093/ve/veaf089 (PMC12640543; doi:10.1093/ve/veaf089)

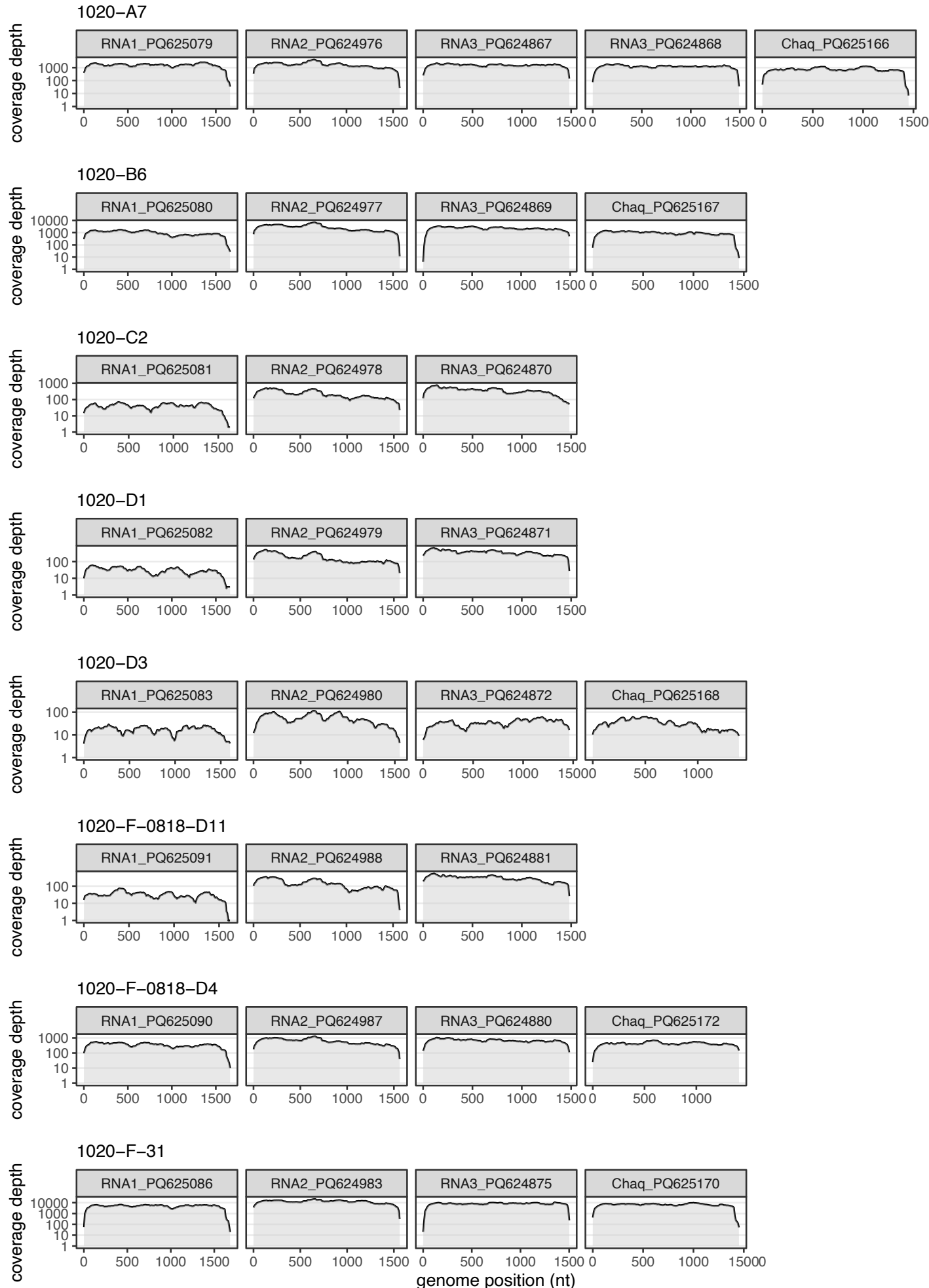

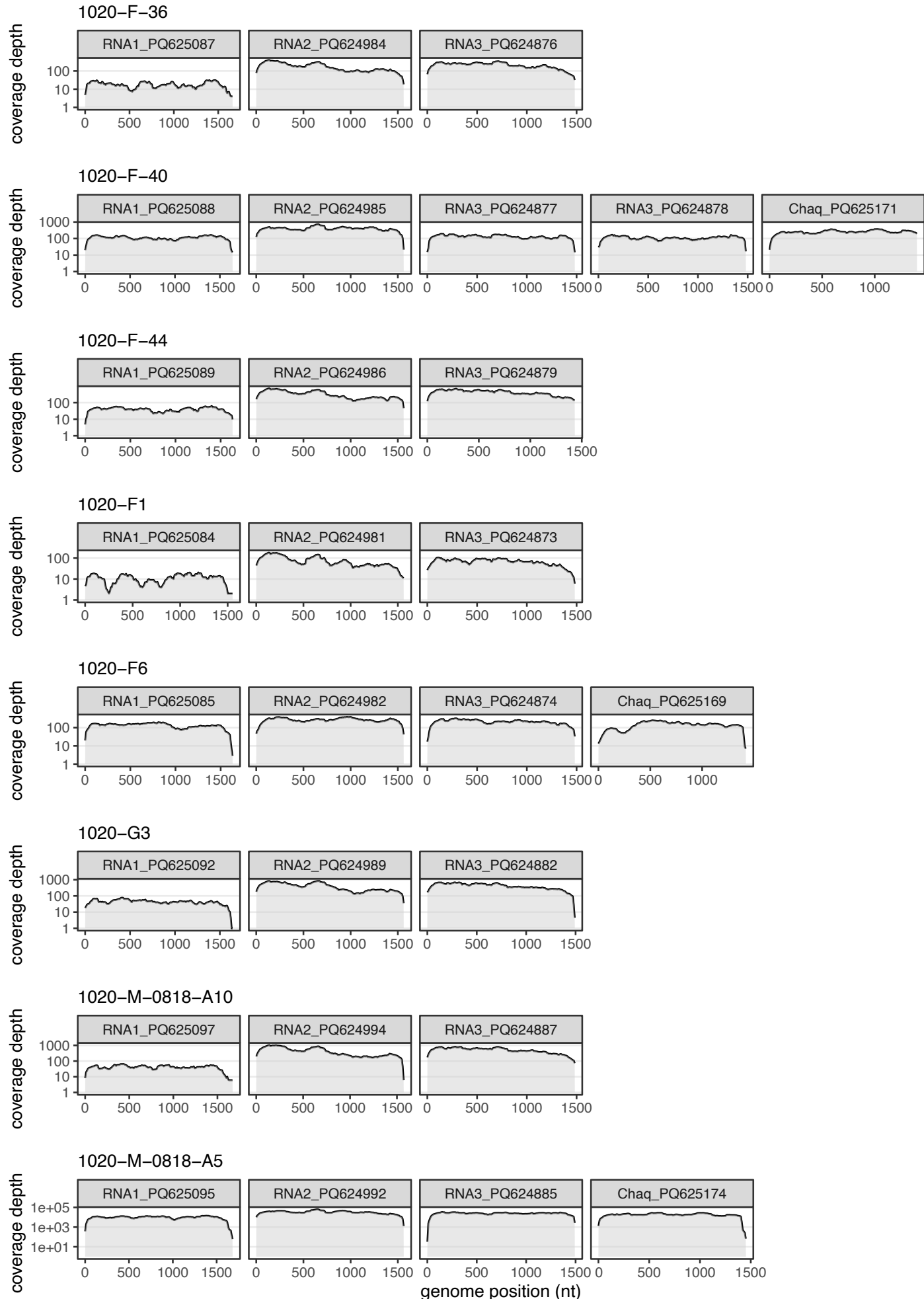

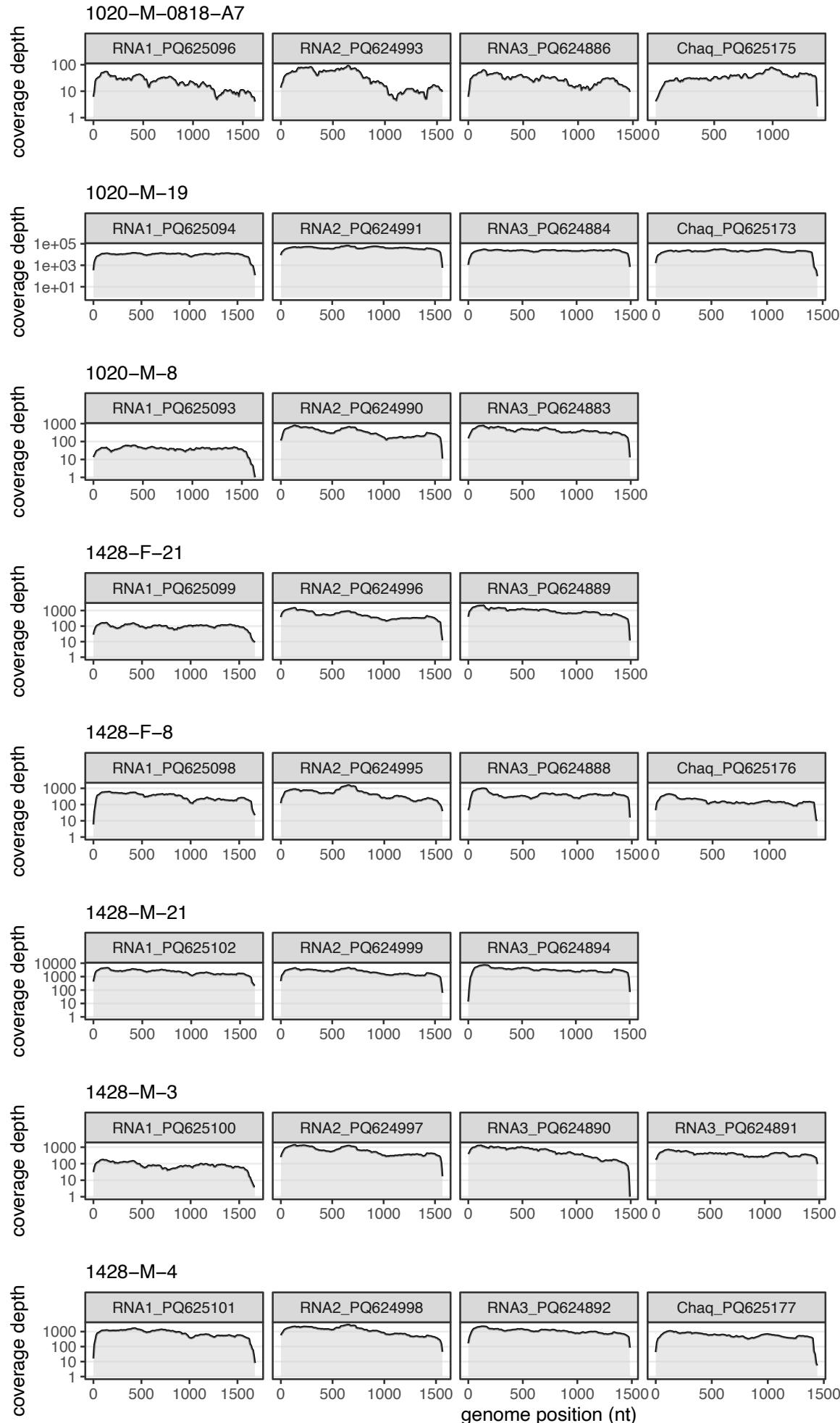

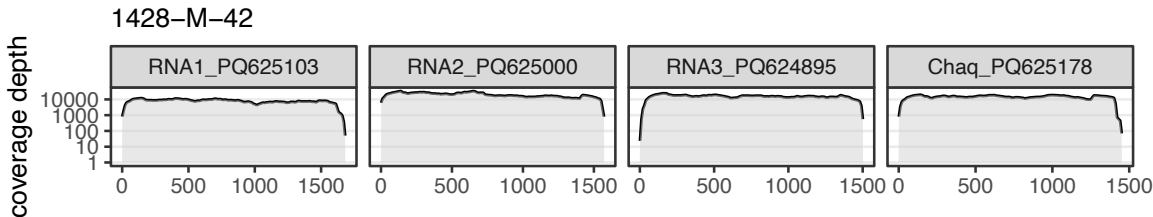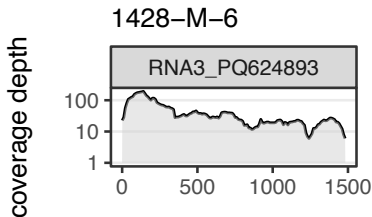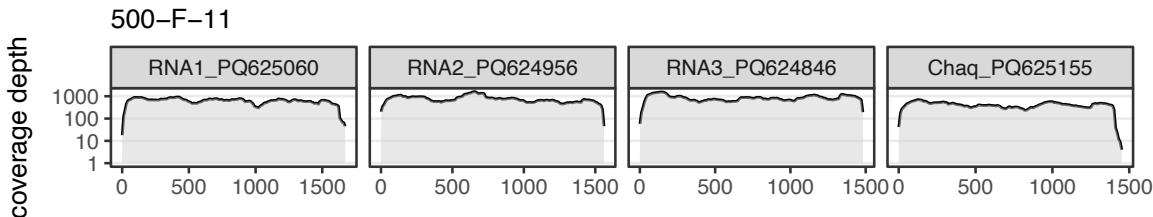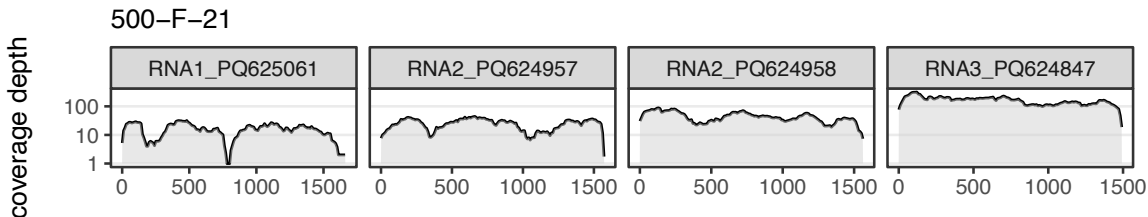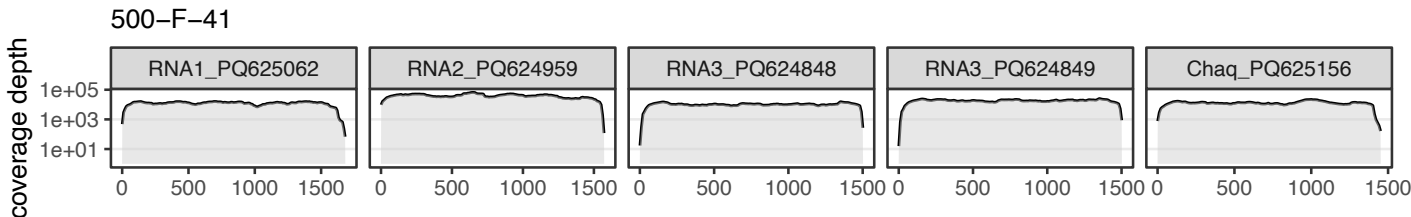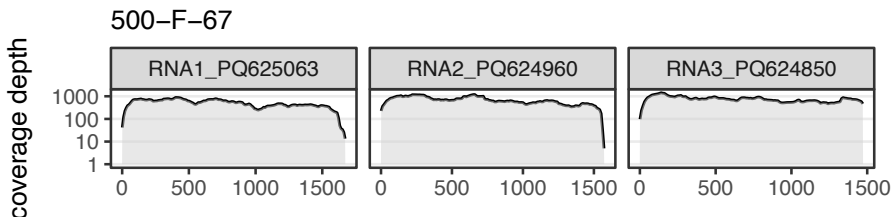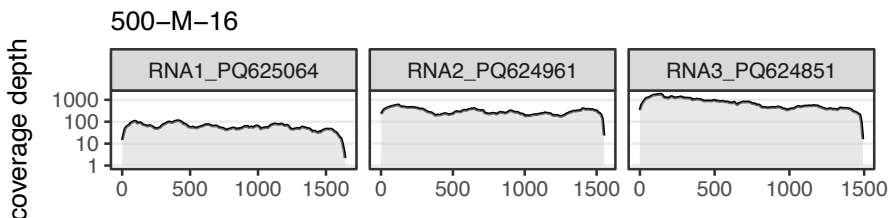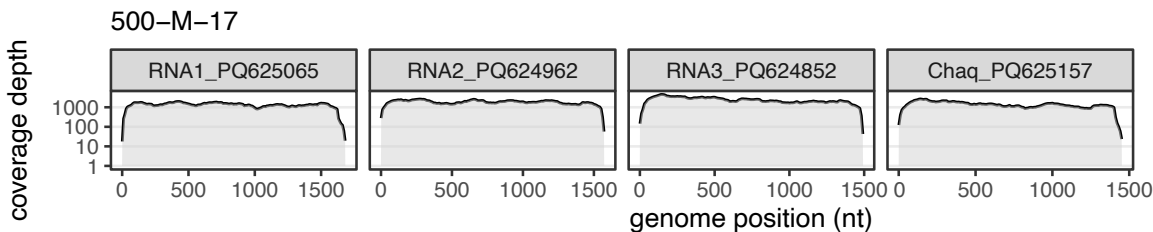

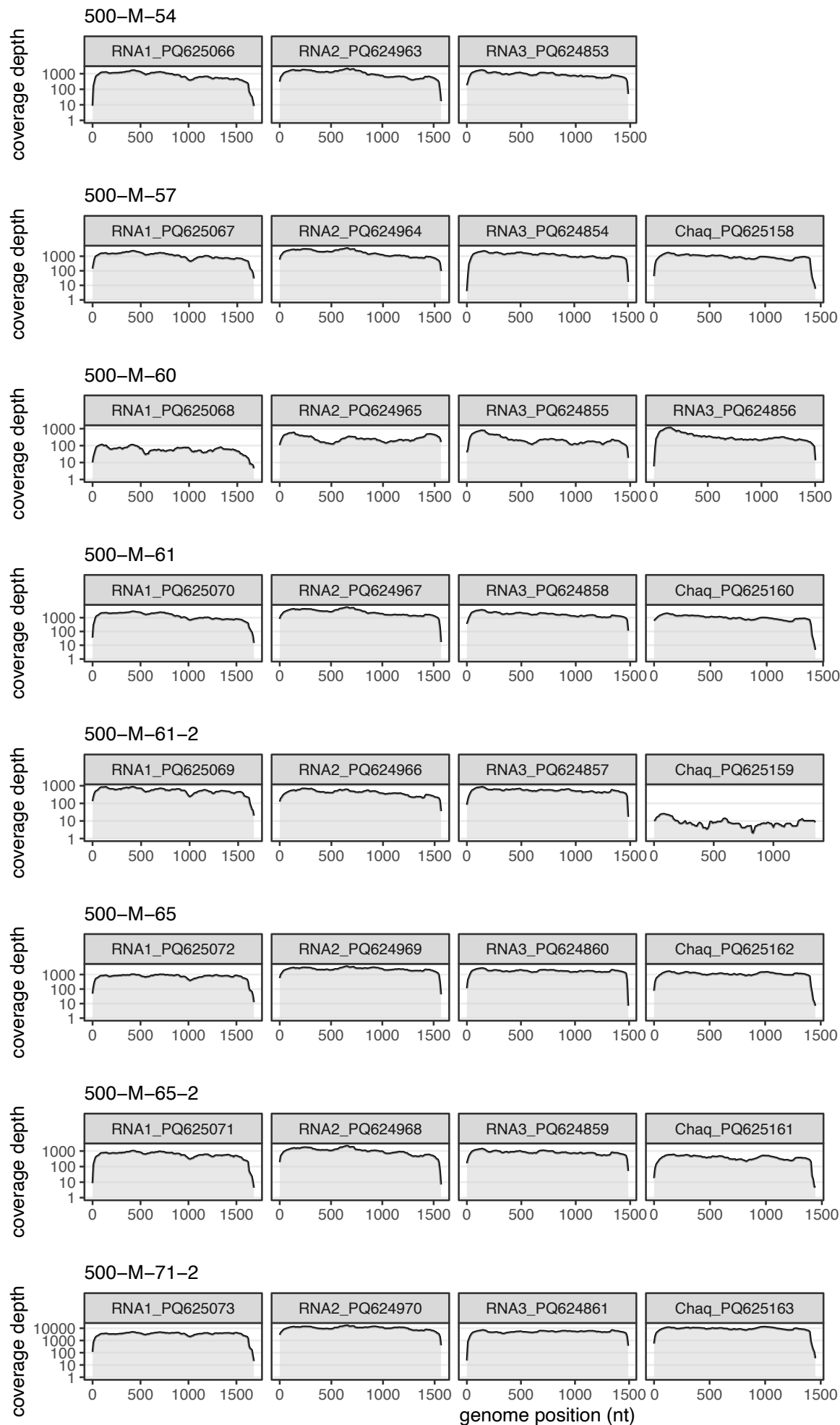

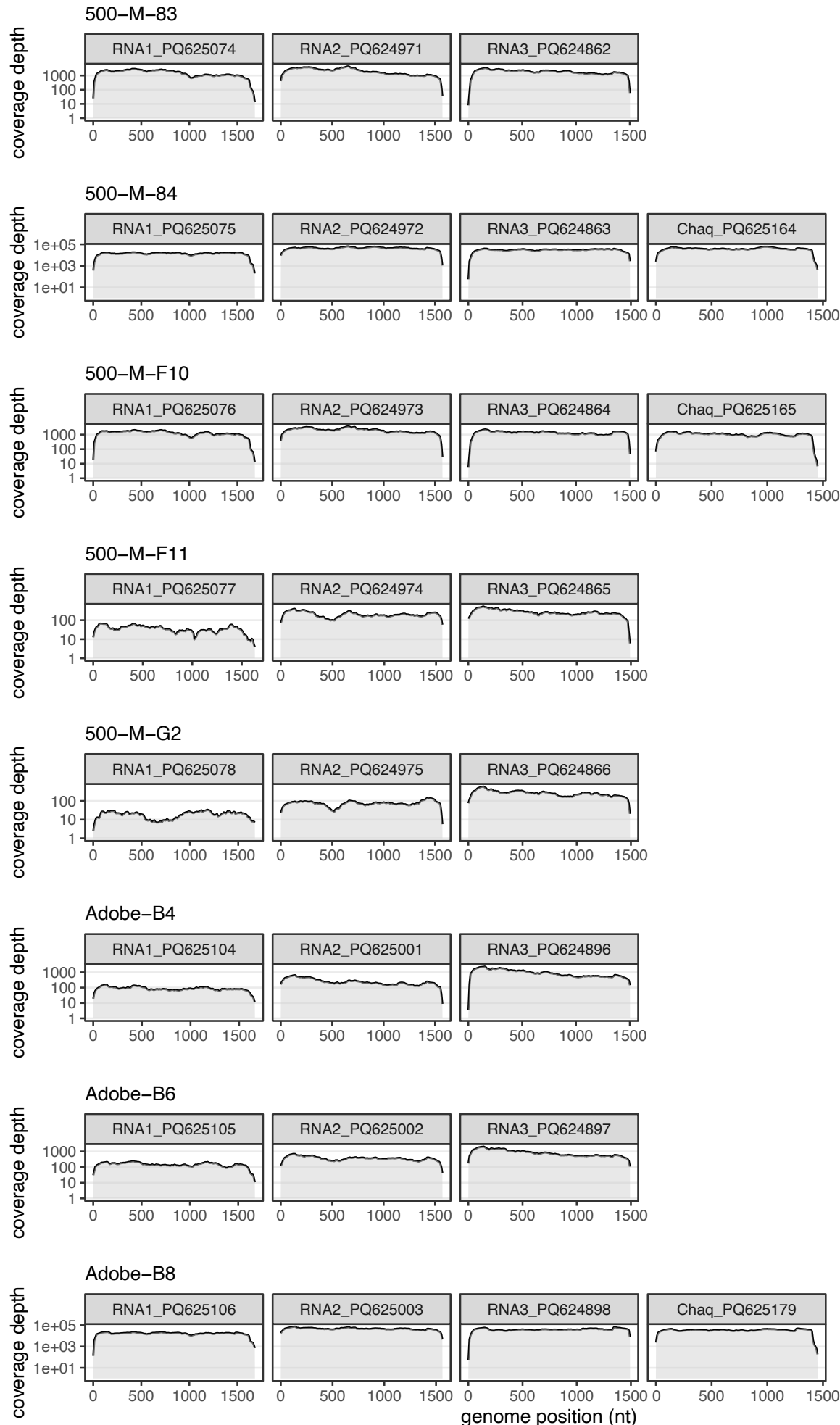

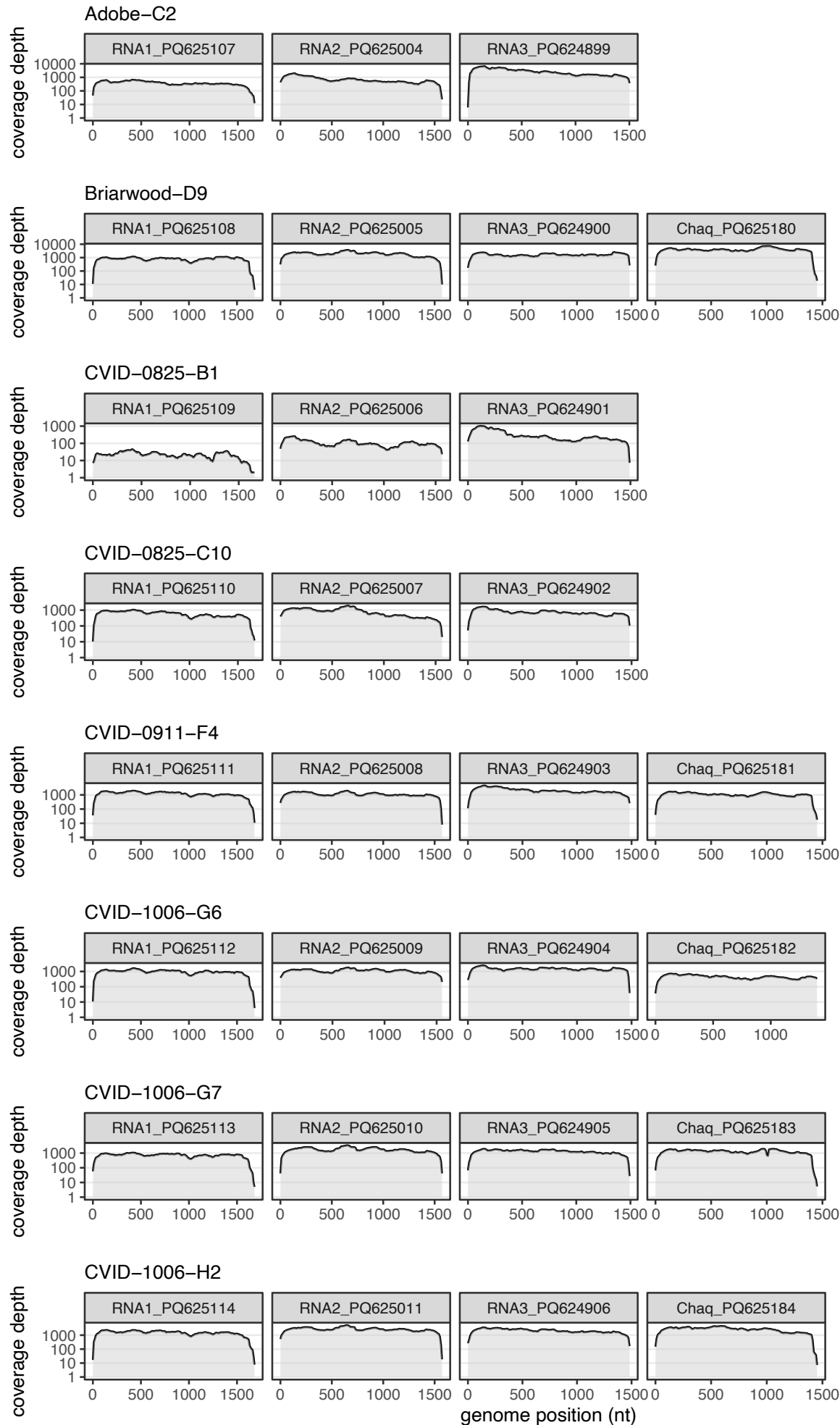

### CVID-1006-H3

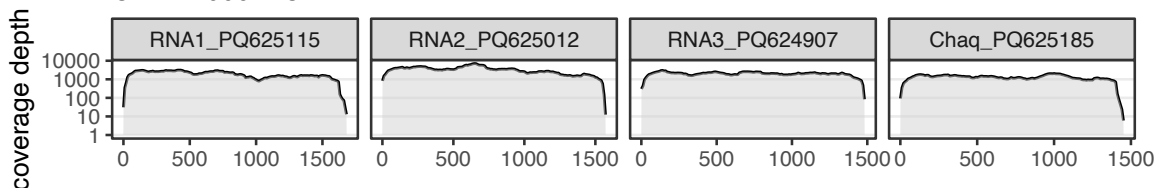

### James-A4

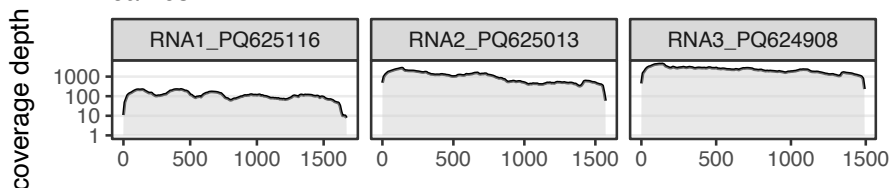

### James-A5

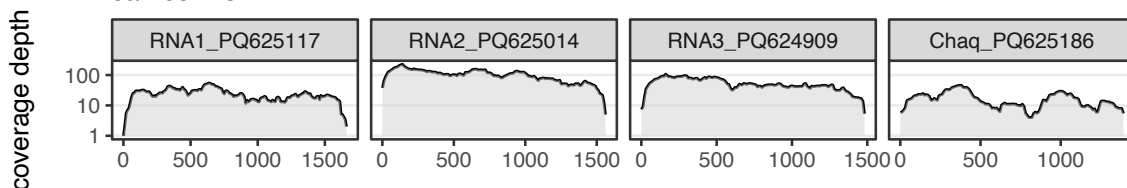

### ME-F-1

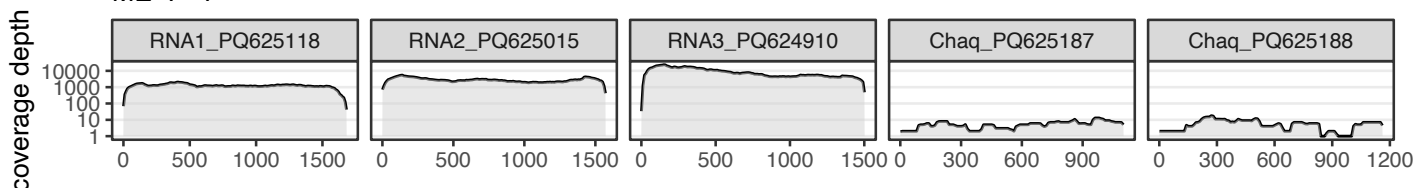

### ME-F-2

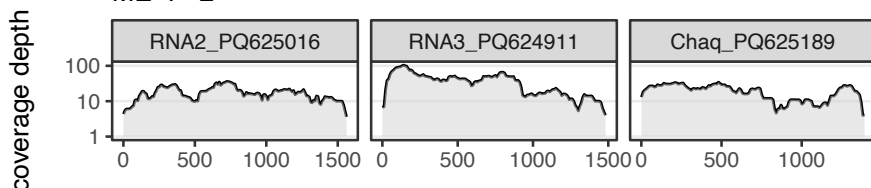

### ME-F-3

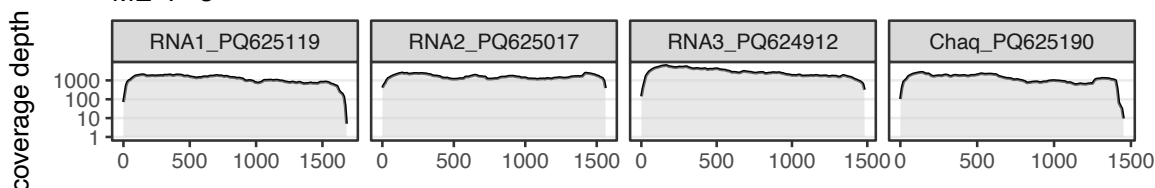

### ME-F-9

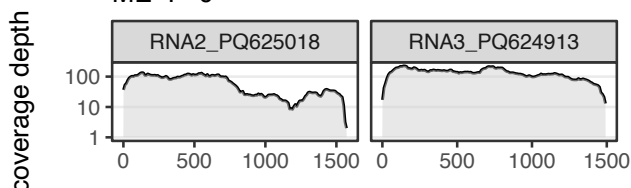

### ME-M-1

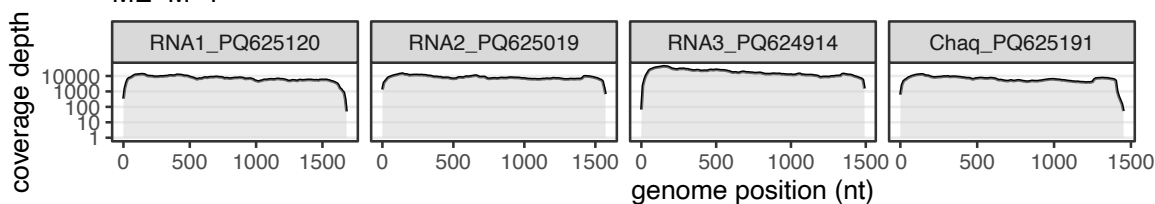

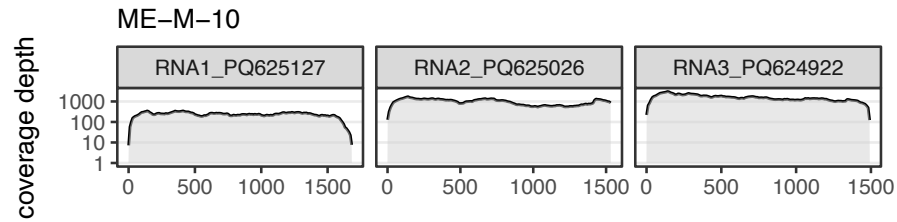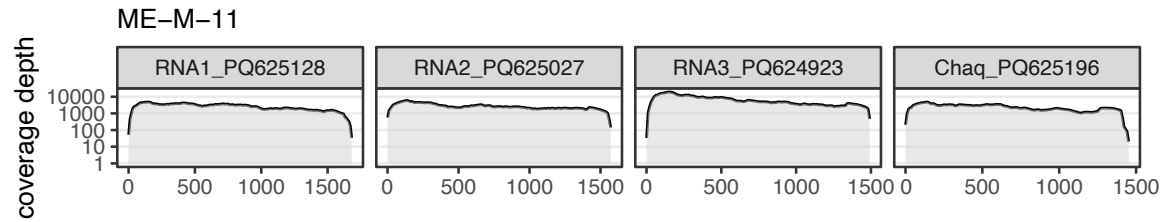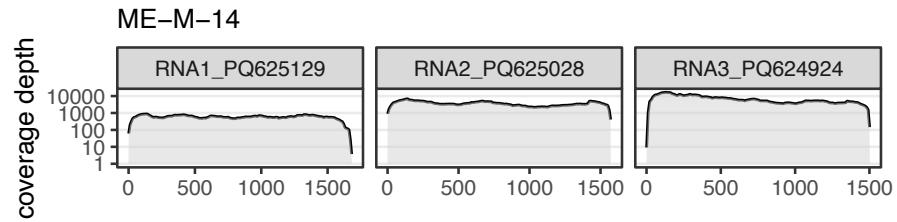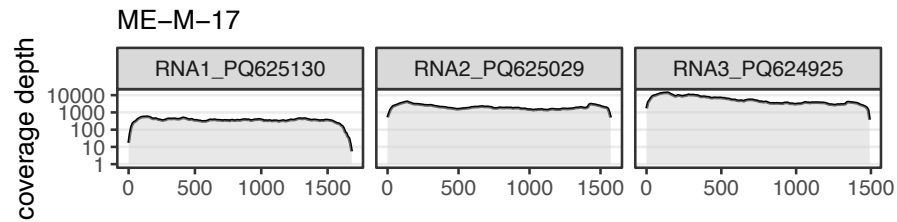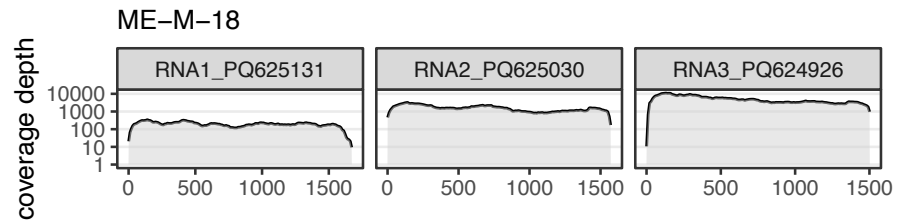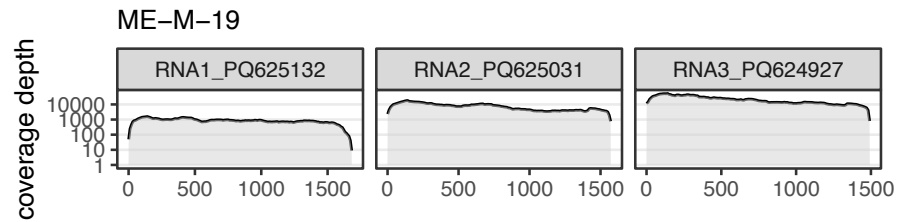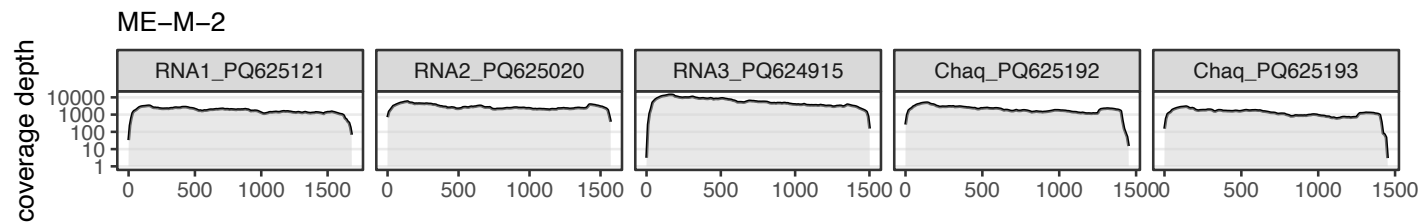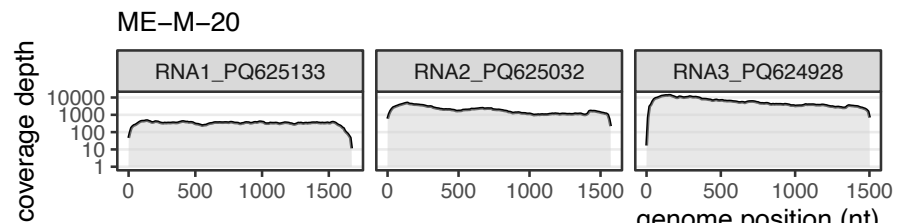

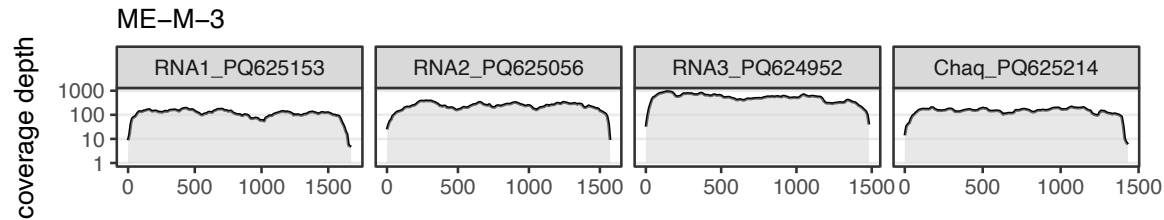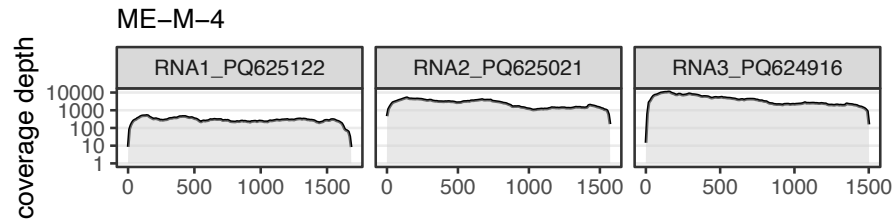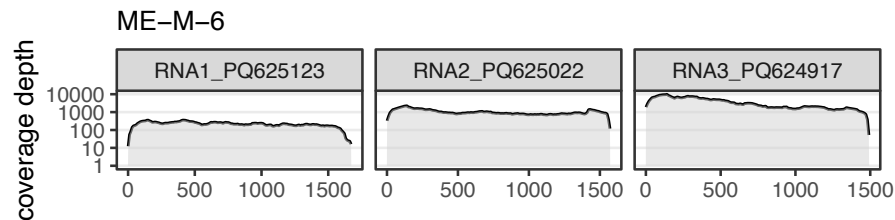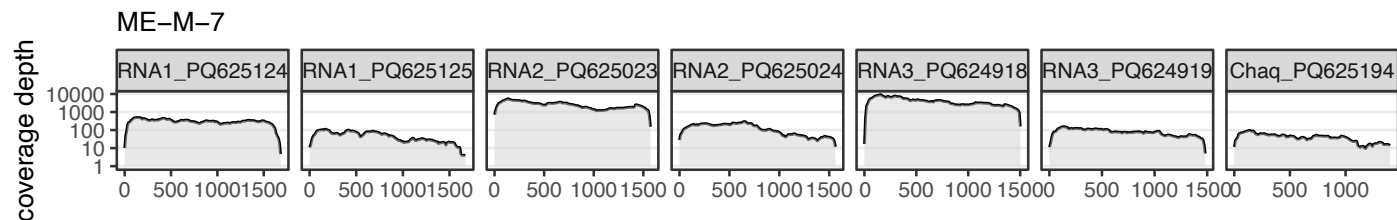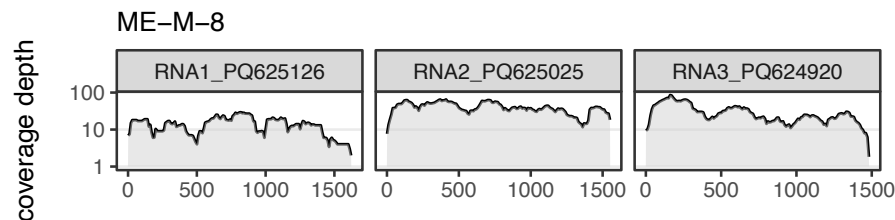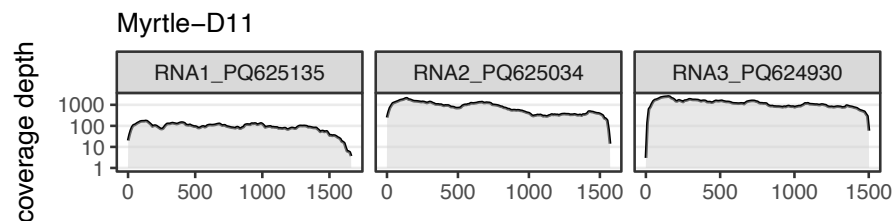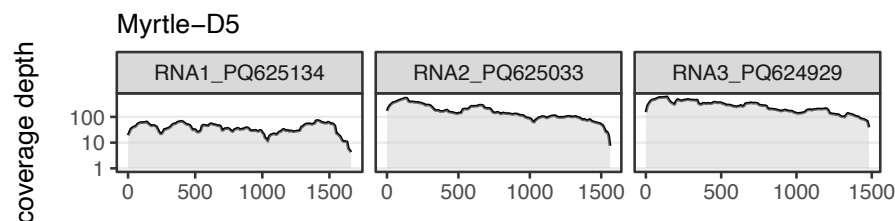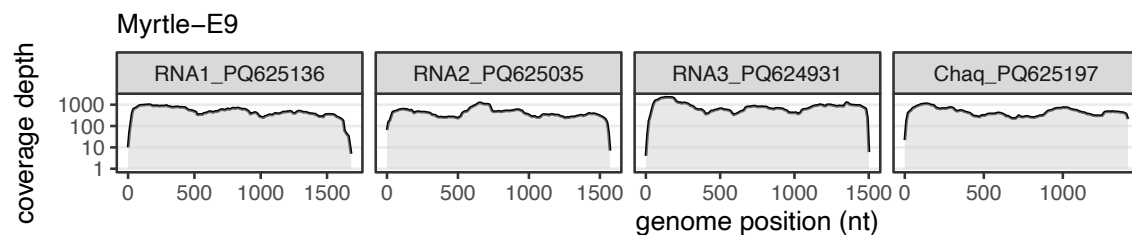

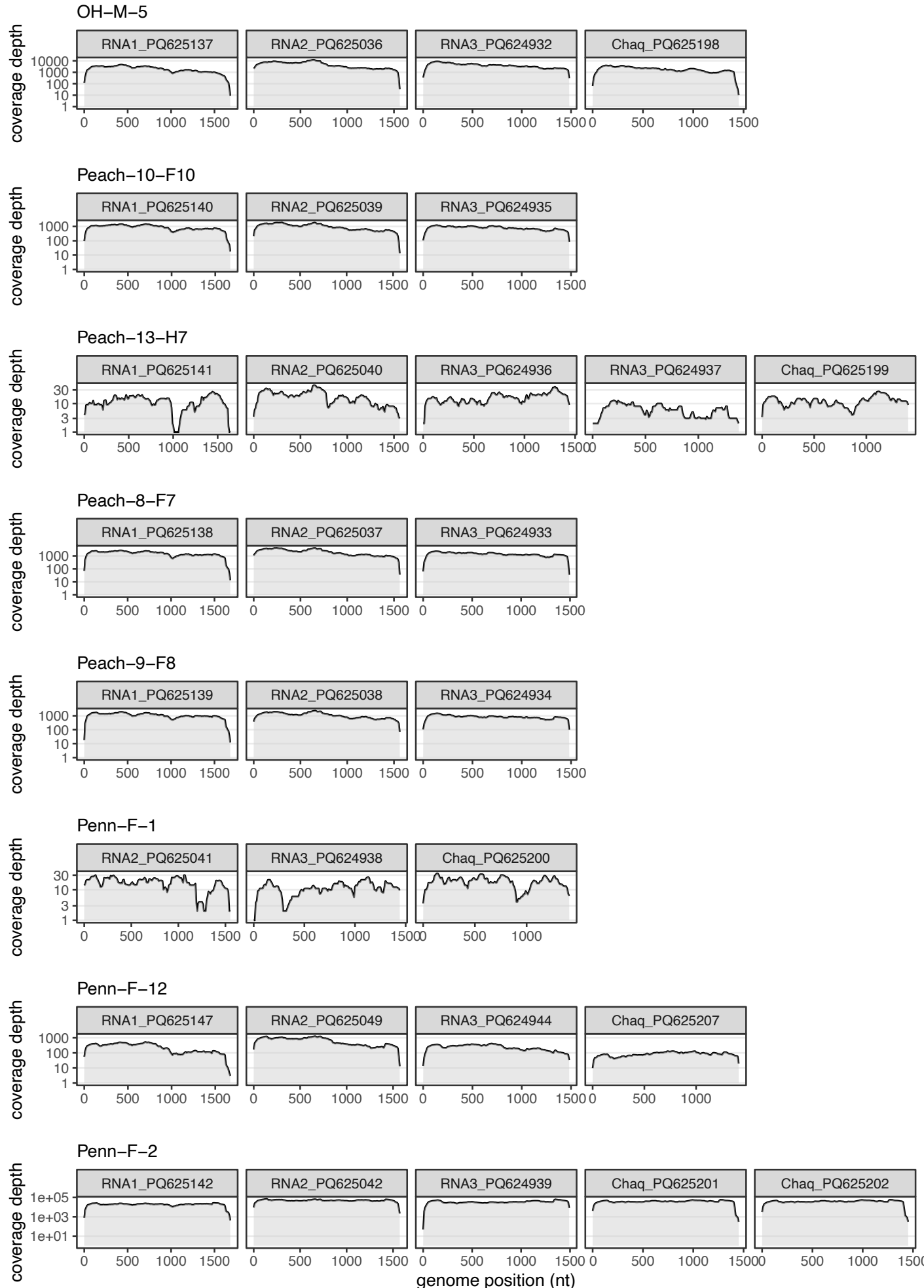

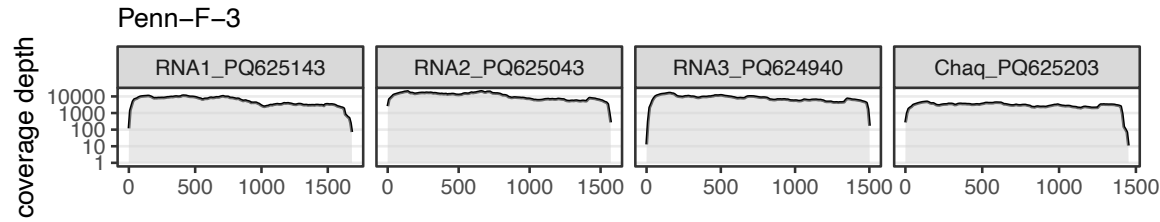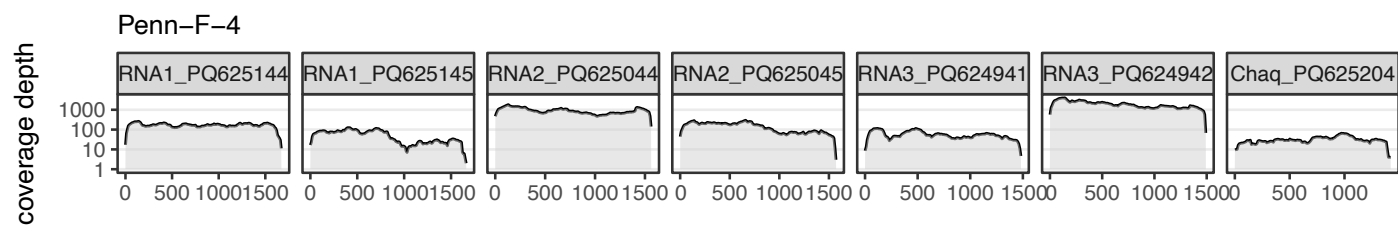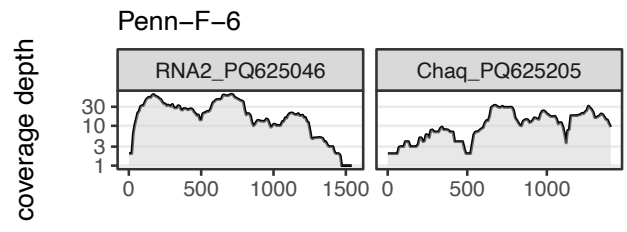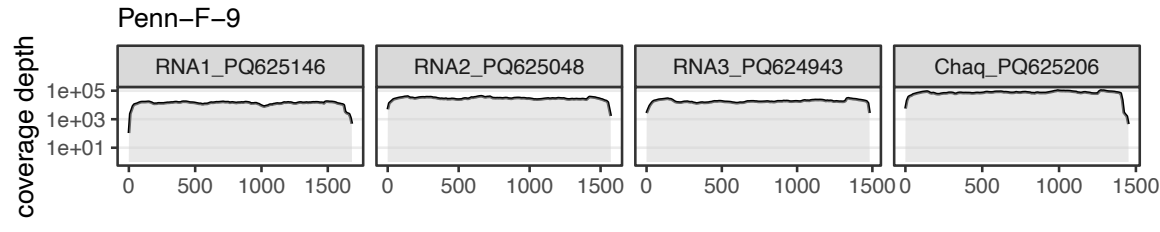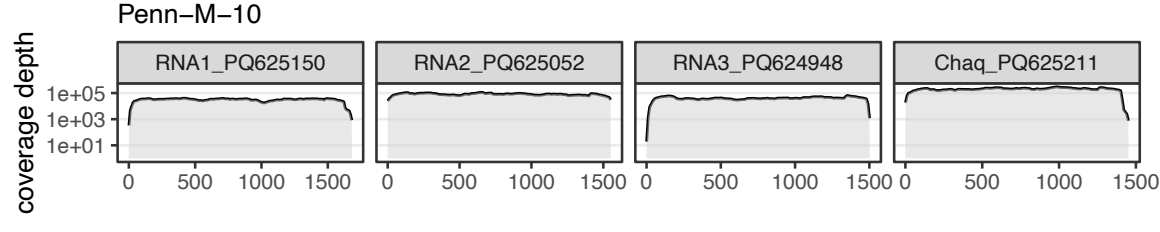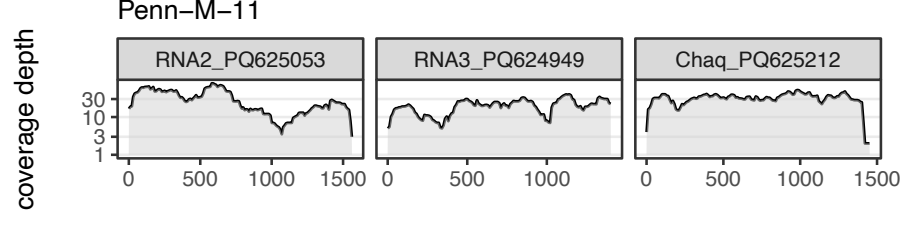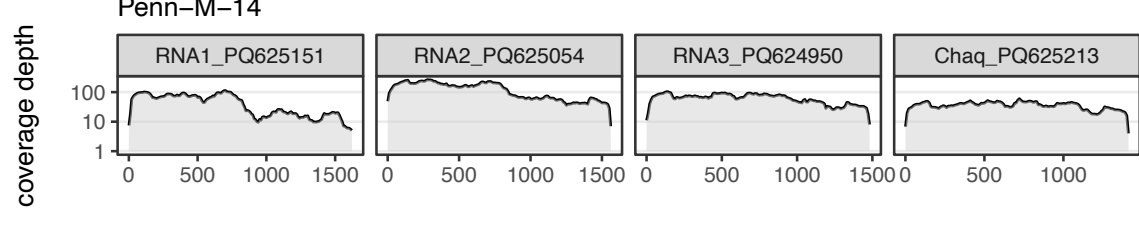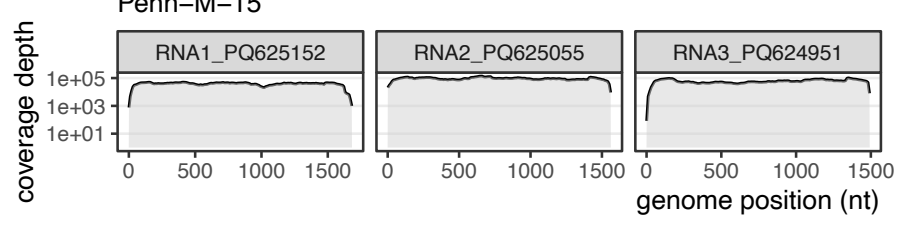

### Penn-M-2

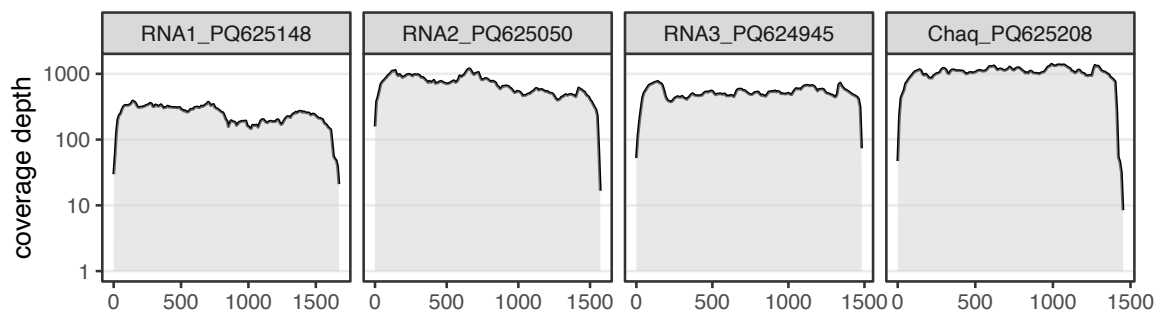

### Penn-M-8

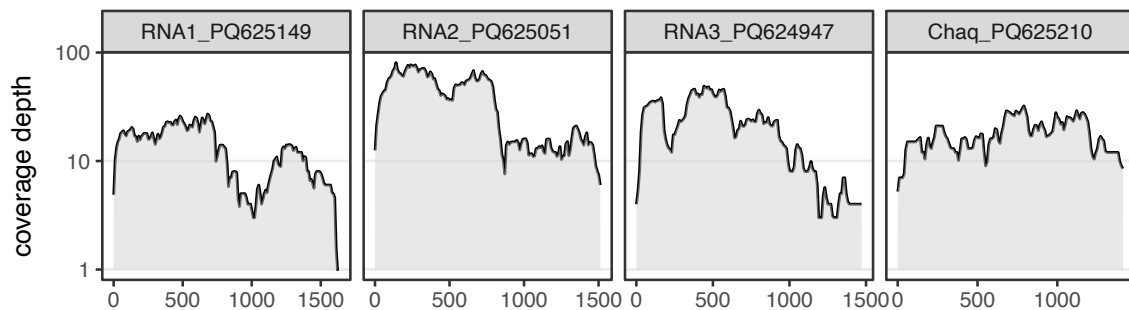

### mel-20-TD-3

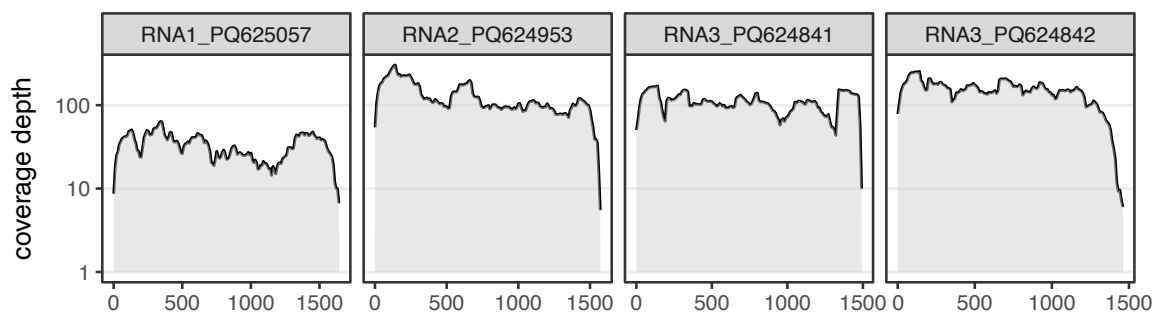

### mel-21-MDS-9

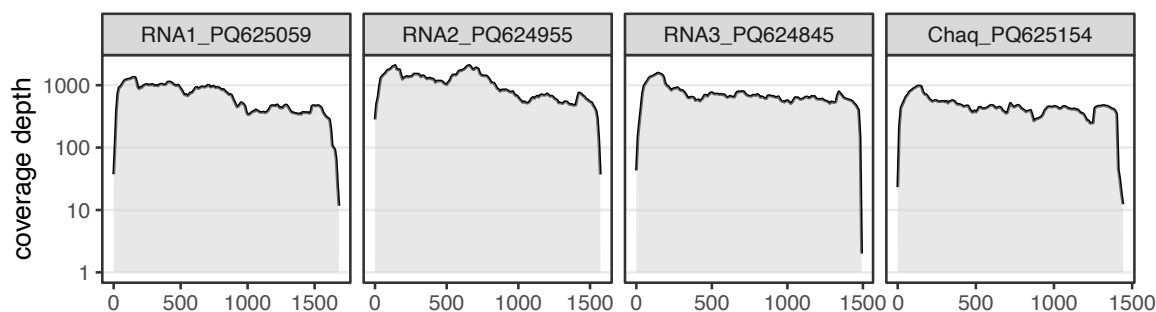

### sim-20-TD-4

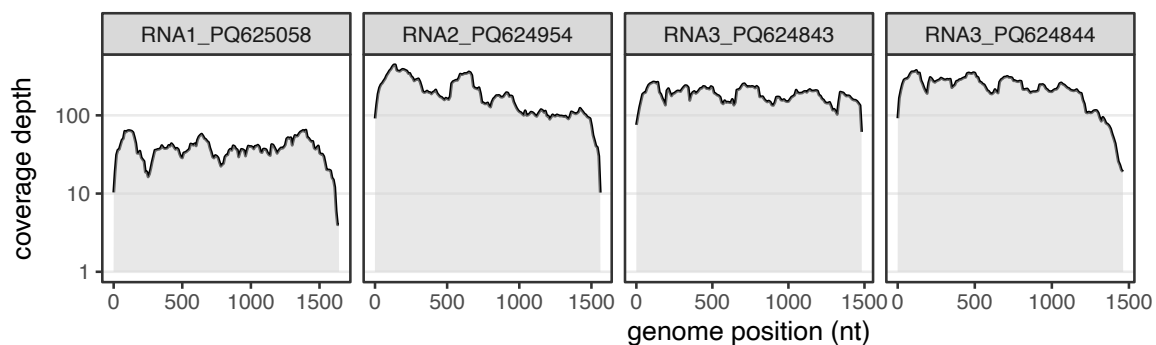

Supplement: Supplemental_Figure_1_coverage_plots_veaf089 [file supplemental_figure_1_coverage_plots_veaf089.pdf]

A

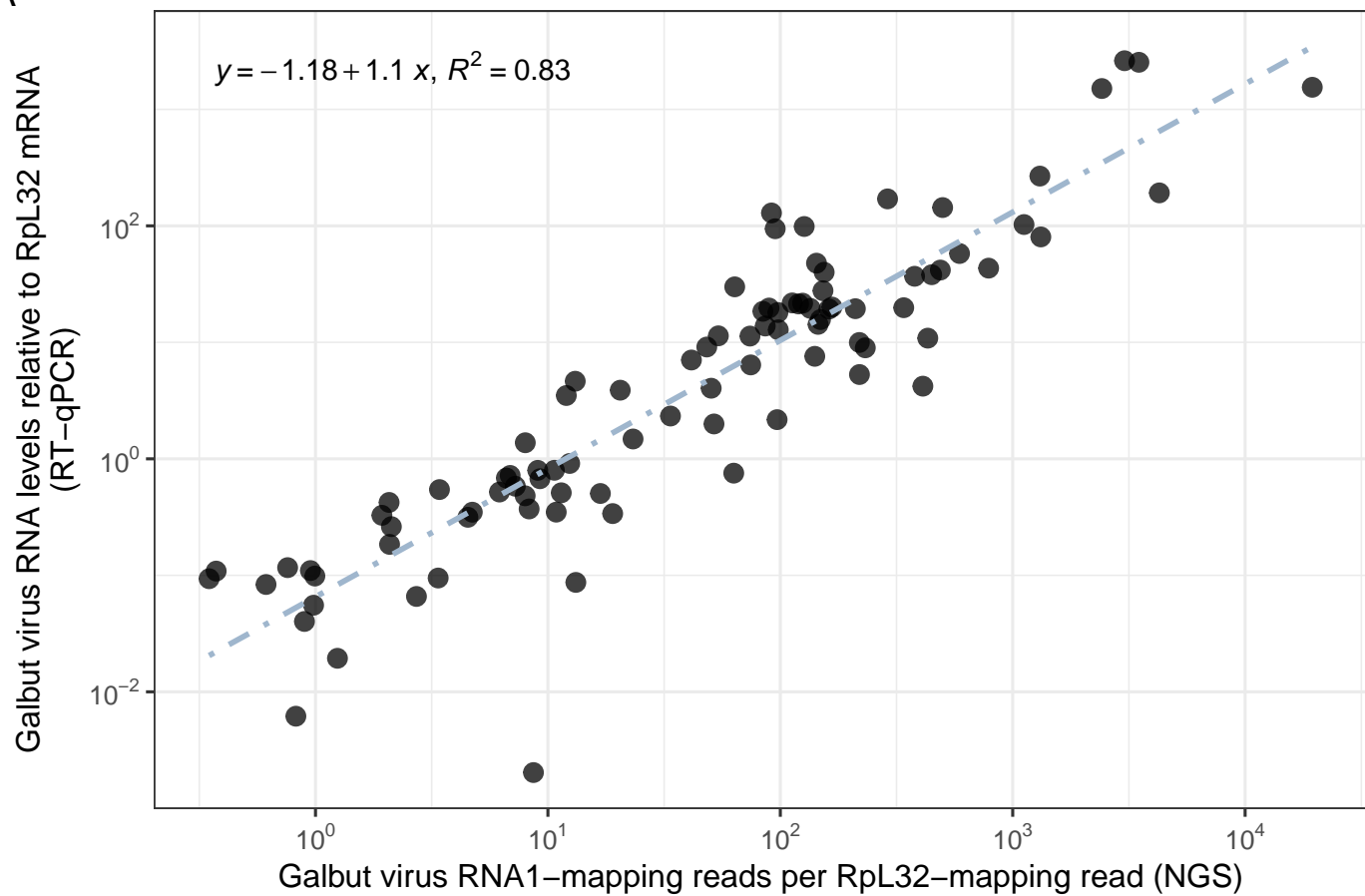

B

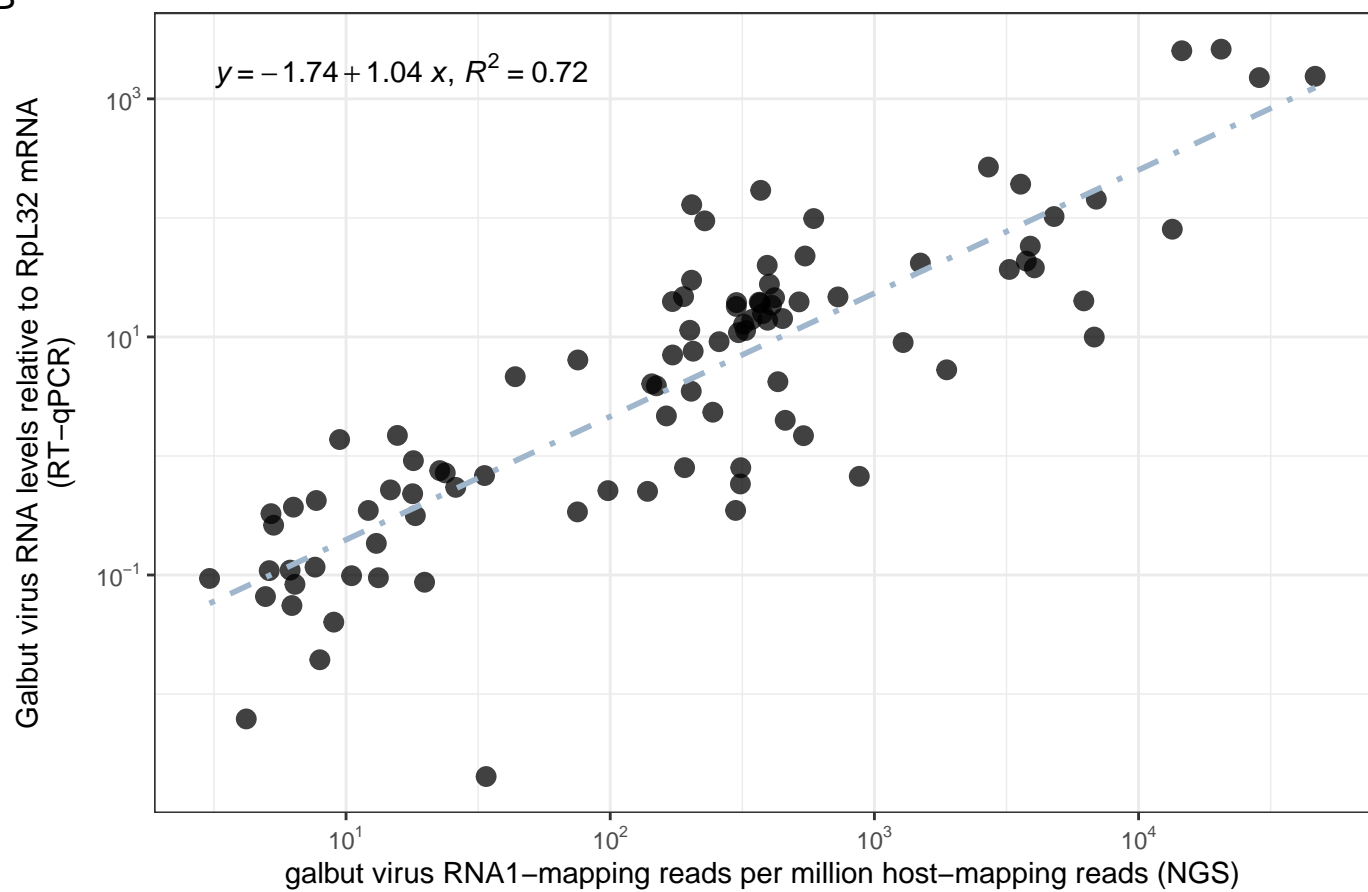

Supplement: Supplemental_Figure_2_qPCR_vs_NGS_quantification_veaf089 [file supplemental_figure_2_qpcr_vs_ngs_quantification_veaf089.pdf]

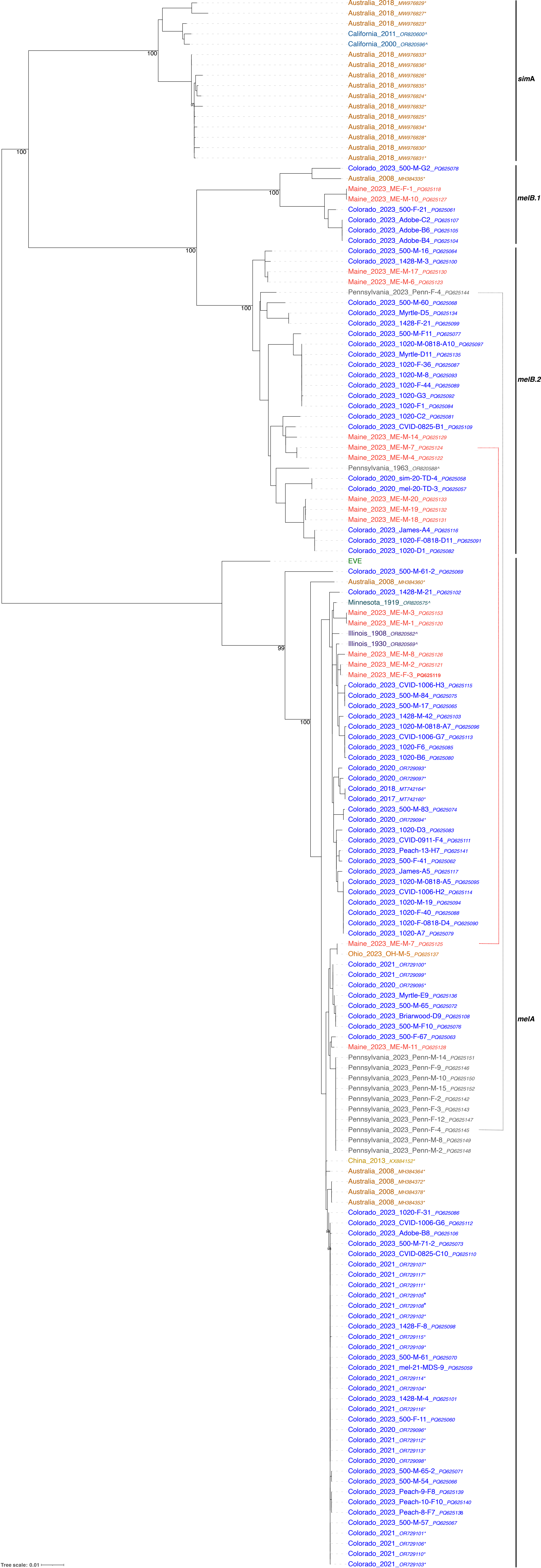

Supplement: Supplemental_figure_3_full_RNA1_tree_veaf089 [file supplemental_figure_3_full_rna1_tree_veaf089.pdf]

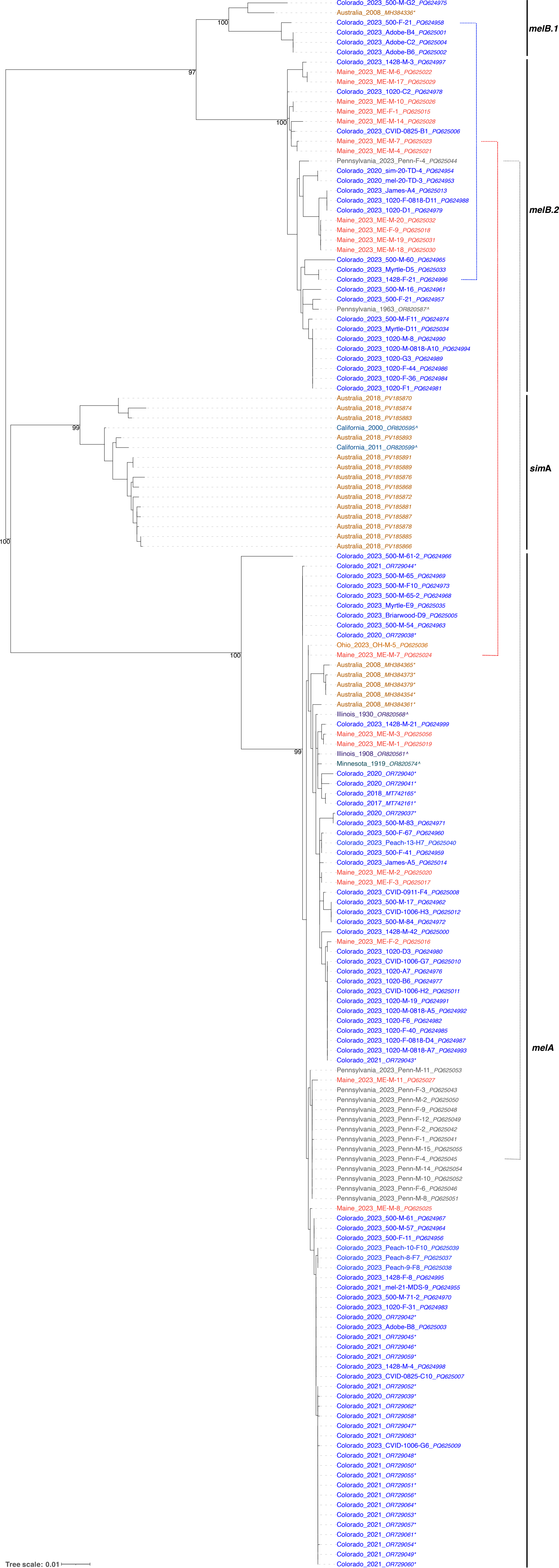

Supplement: Supplemental_figure_4_full_RNA2_tree_veaf089 [file supplemental_figure_4_full_rna2_tree_veaf089.pdf]

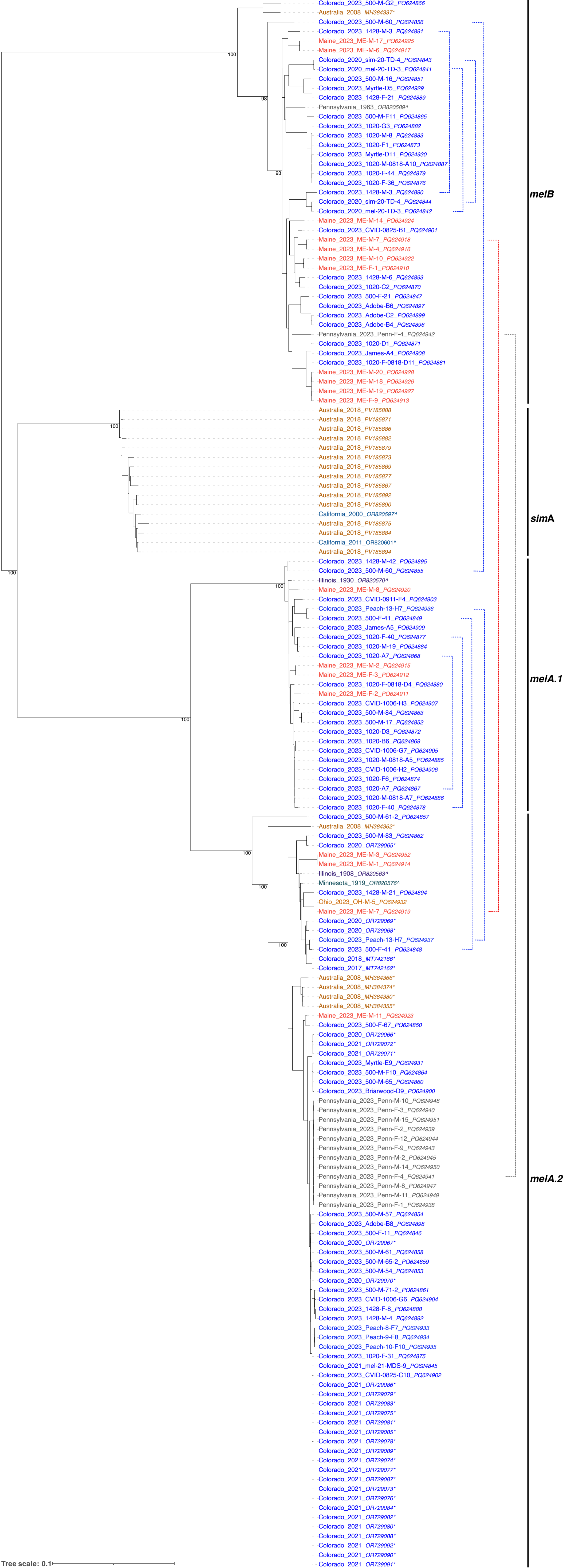

Supplement: Supplemental_figure_5_full_RNA3_tree_veaf089 [file supplemental_figure_5_full_rna3_tree_veaf089.pdf]

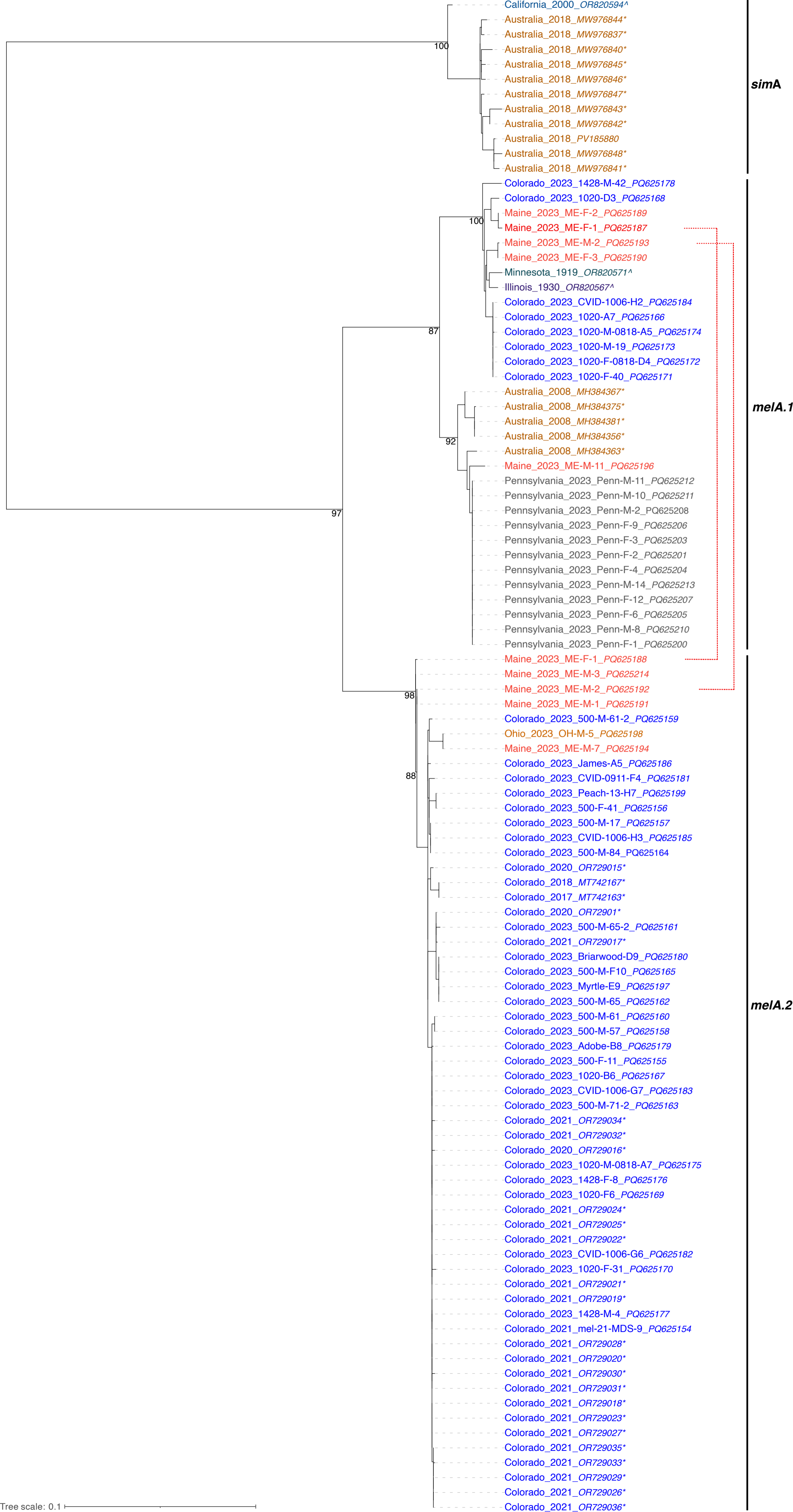

Supplement: Supplemental_figure_6_full_chaq_tree_veaf089 [file supplemental_figure_6_full_chaq_tree_veaf089.pdf]

**A**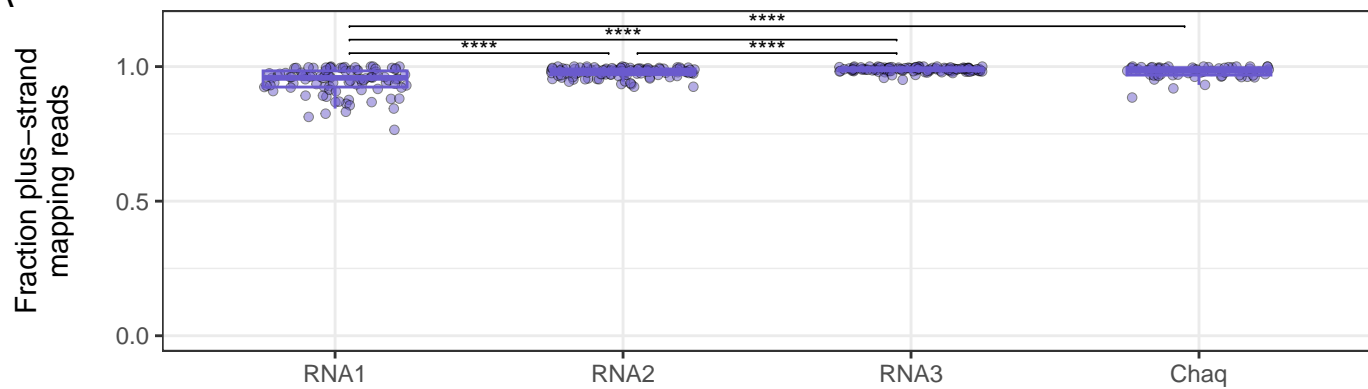**B**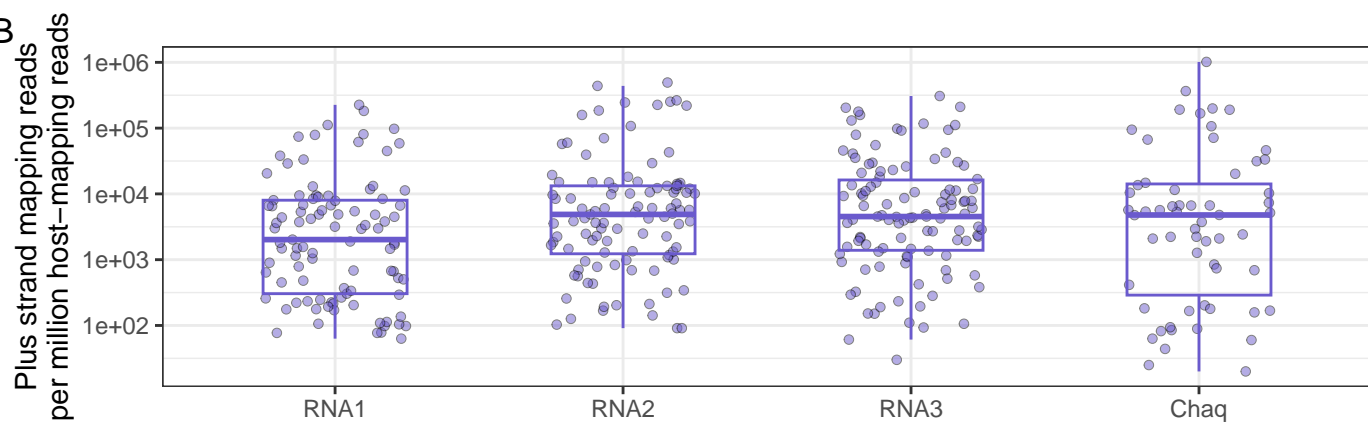**C**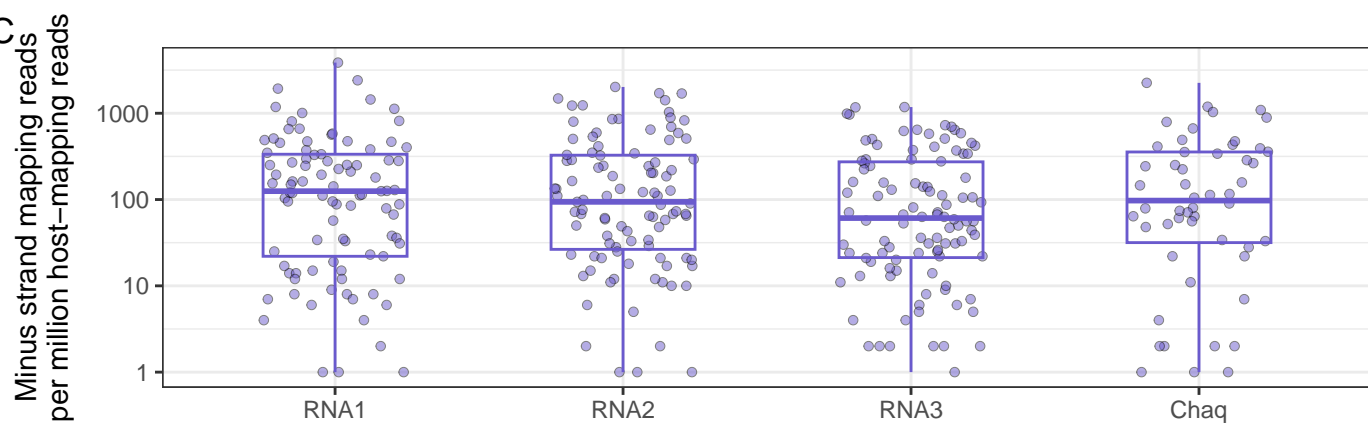**D**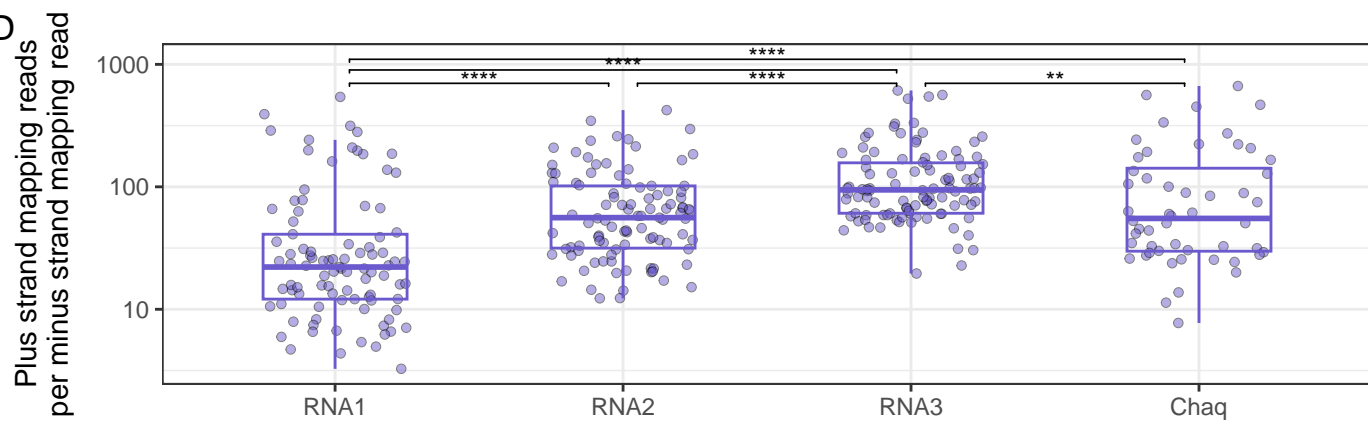

Supplement: Supplemental_figure_7_segment_strand_levels_veaf089 [file supplemental_figure_7_segment_strand_levels_veaf089.pdf]

Number pairwise alignments

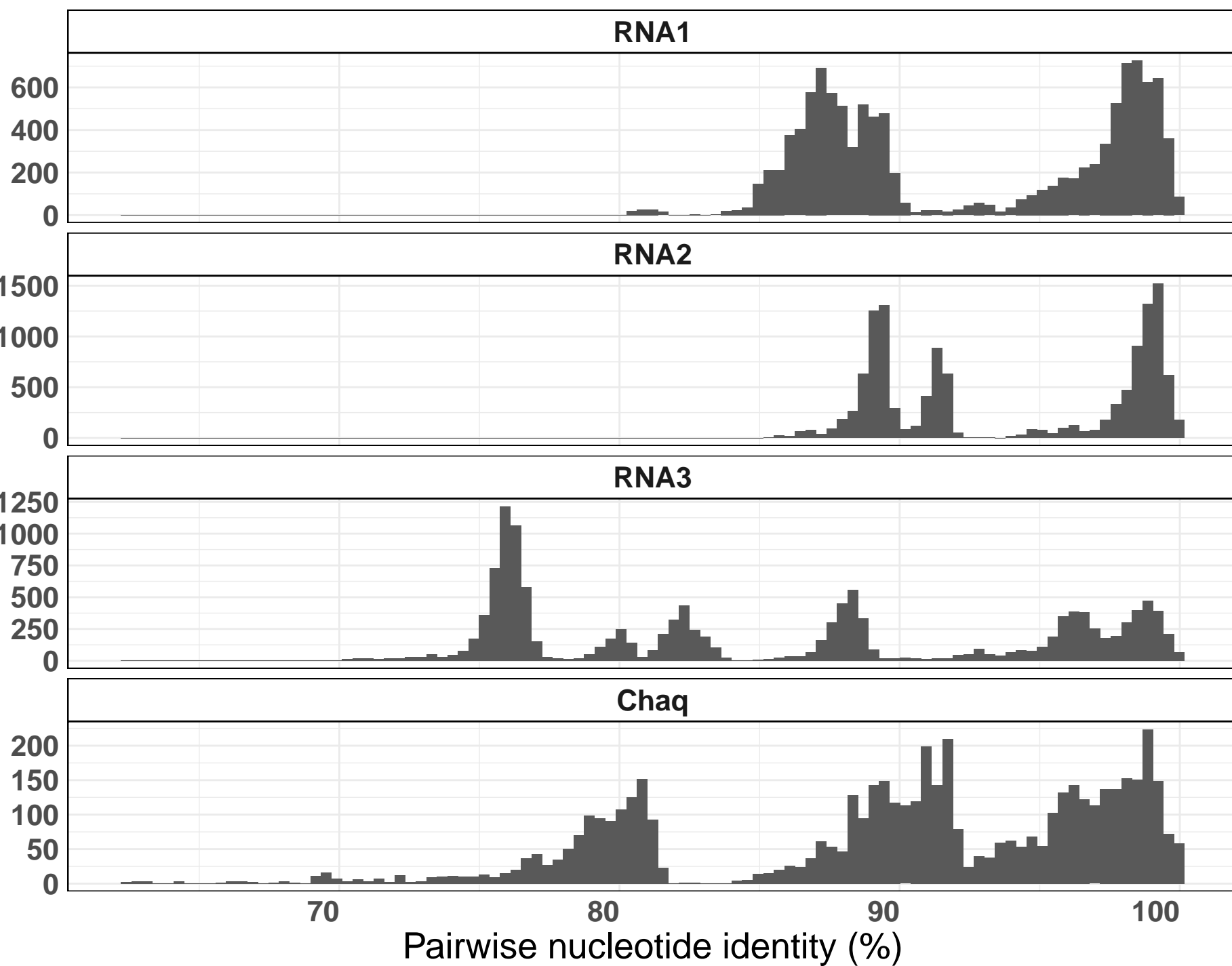

Supplement: Supplemental_figure_8_pairwise_pct_identity_by_segment_veaf089 [file supplemental_figure_8_pairwise_pct_identity_by_segment_veaf089.pdf]

RNA1

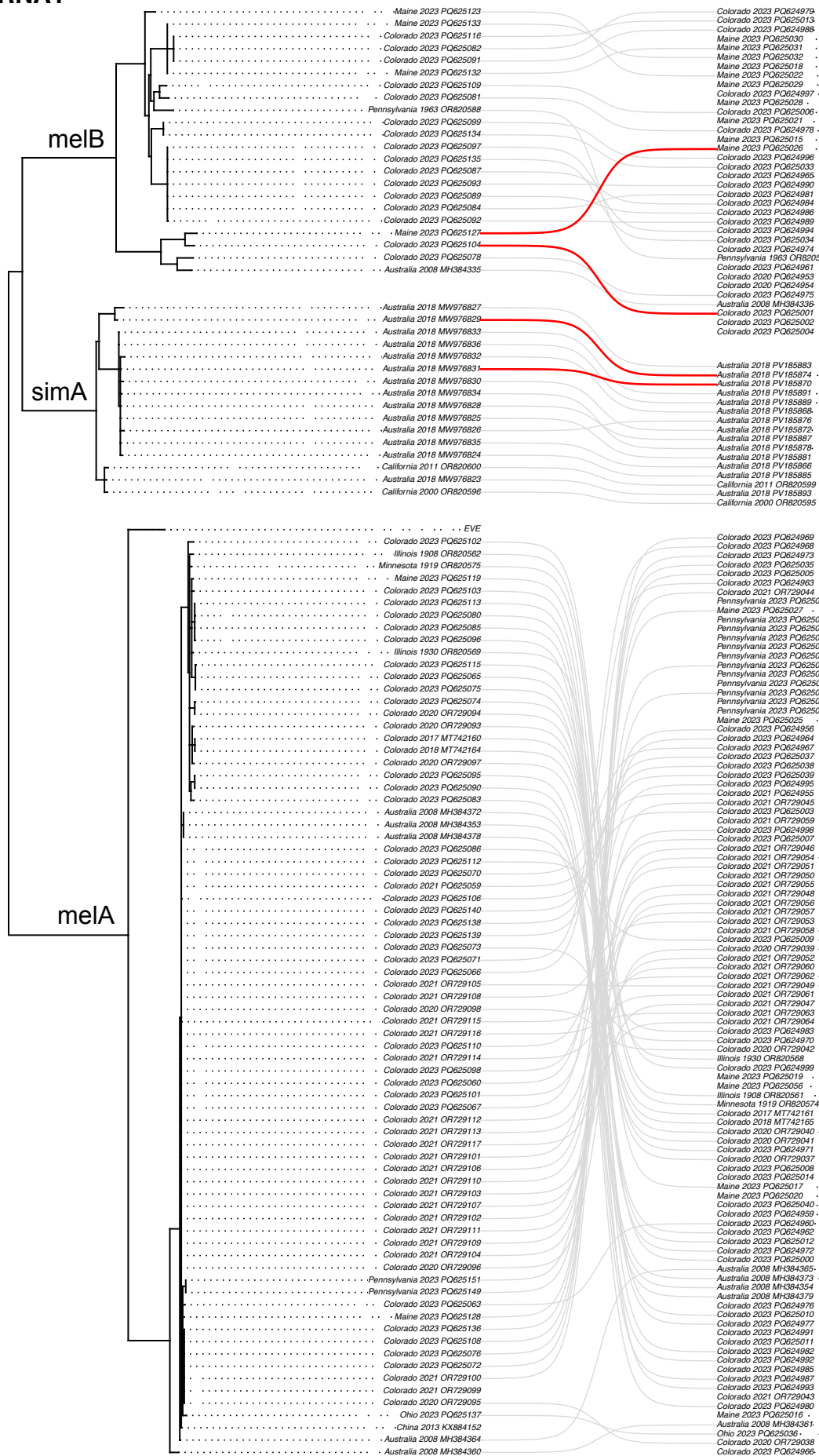

0.025

RNA2

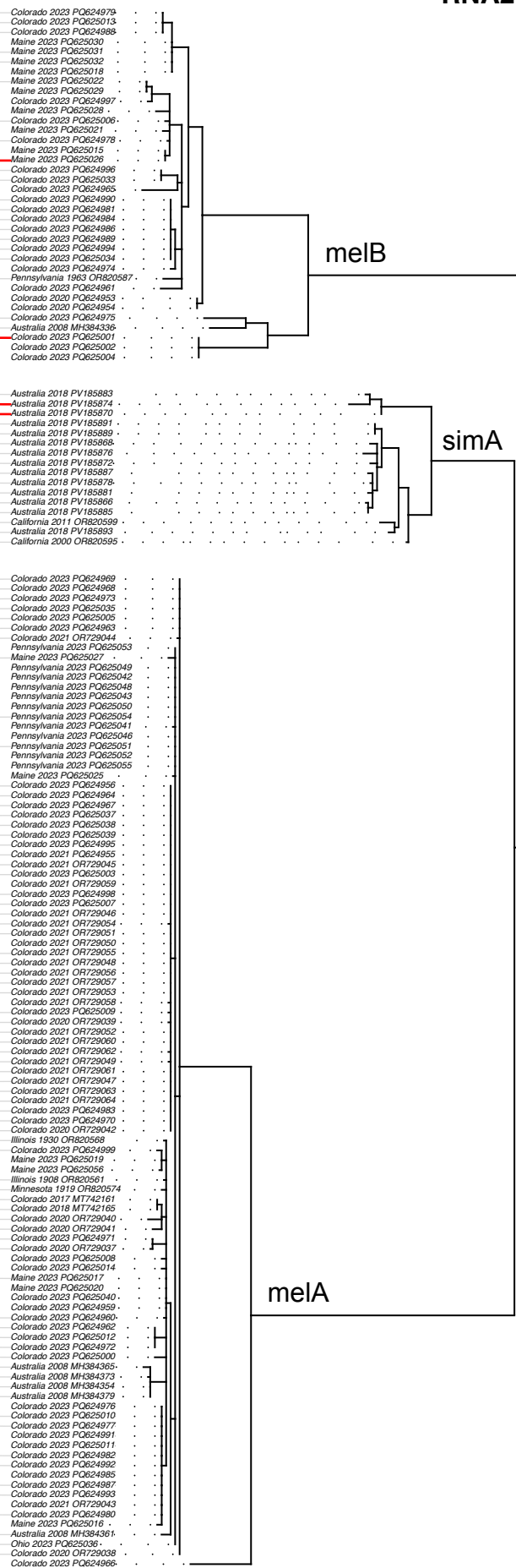

0.025

Supplement: Supplemental_figure_9_RNA1_RNA2_tanglegram_veaf089 [file supplemental_figure_9_rna1_rna2_tanglegram_veaf089.pdf]

RNA2

Chaq

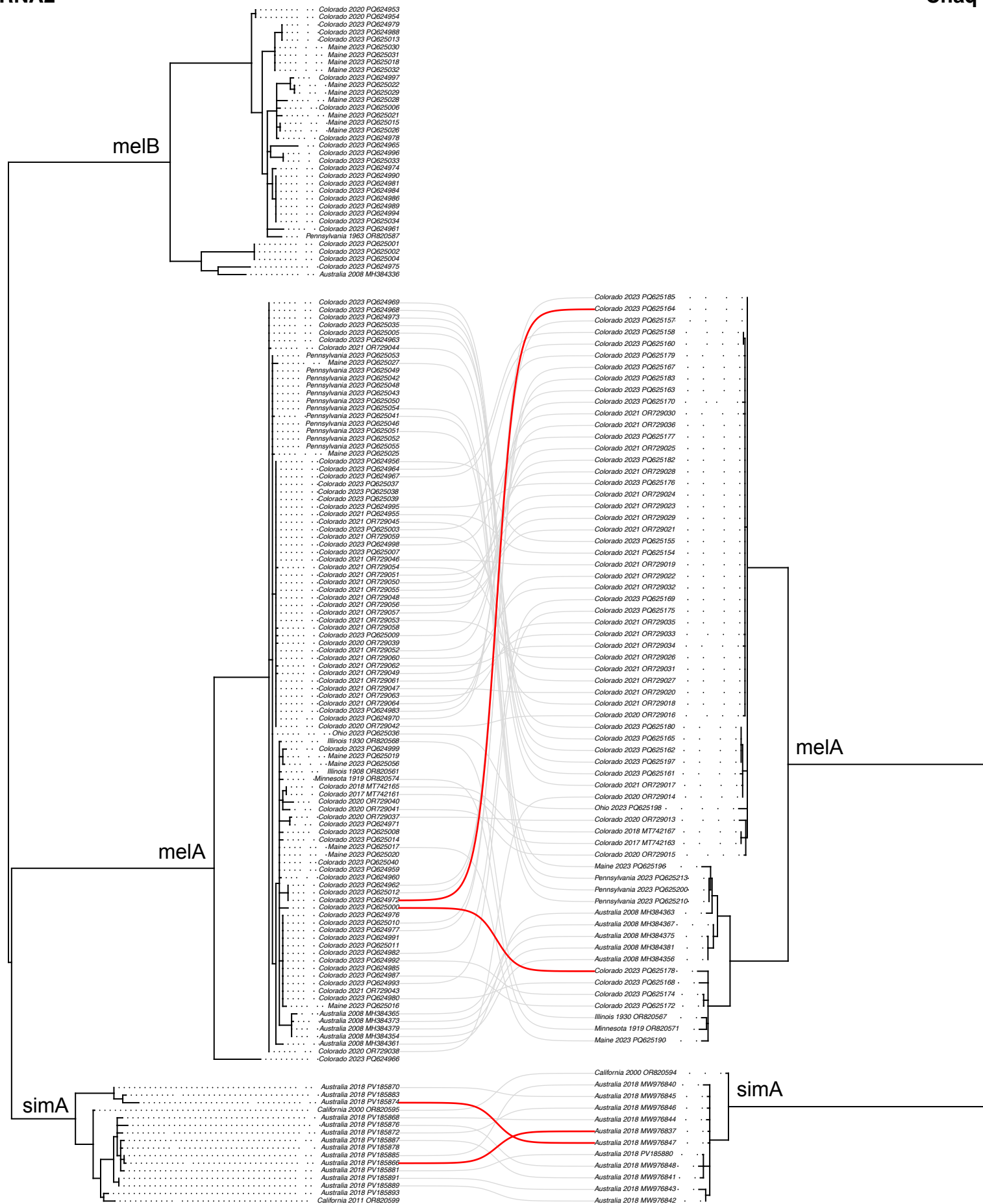

Supplement: Supplemental_figure_11_RNA2_Chaq_tanglegram_veaf089 [file supplemental_figure_11_rna2_chaq_tanglegram_veaf089.pdf]

RNA3

Chaq

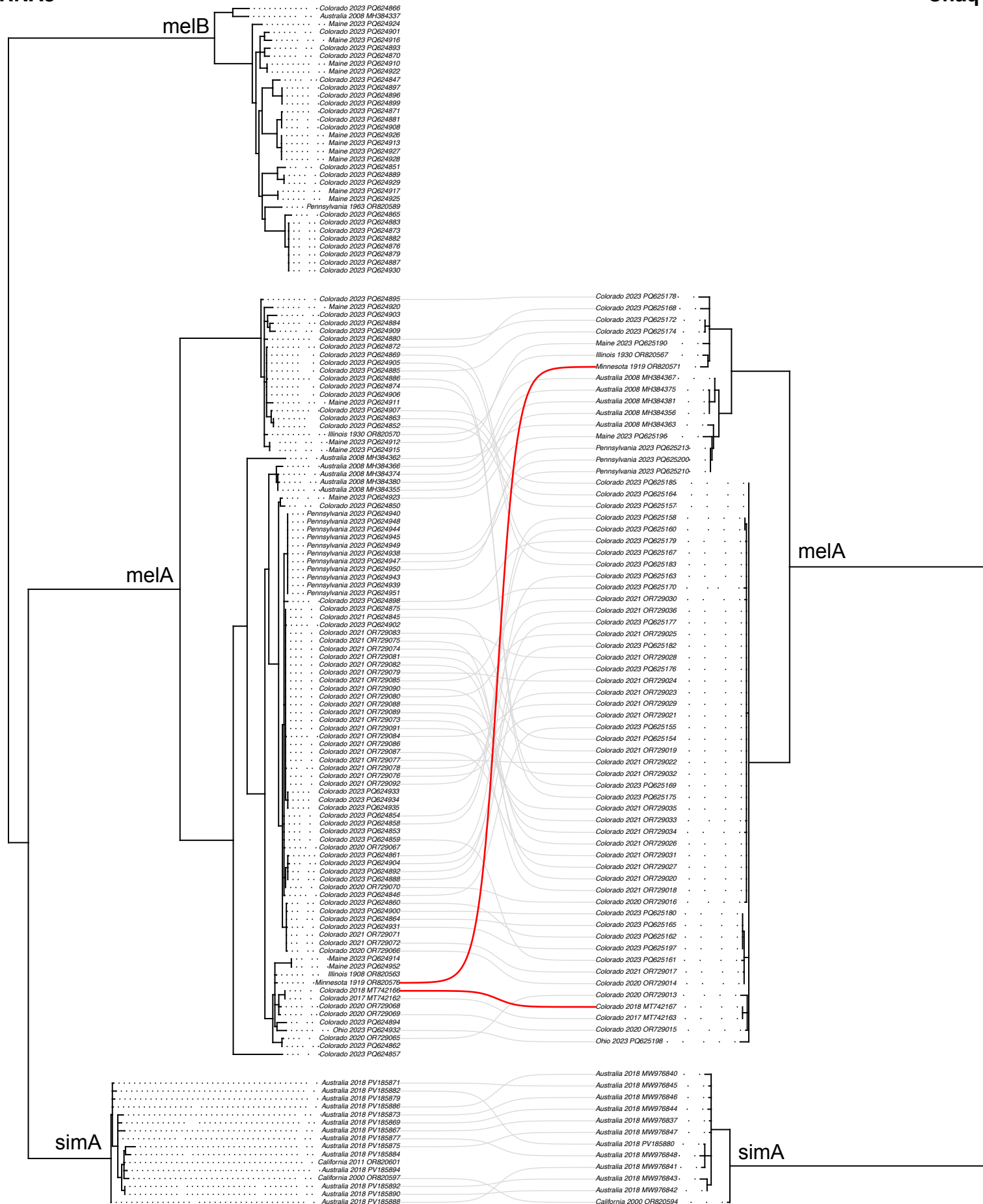

0.025

0.025

Supplement: Supplemental_figure_12_RNA3_Chaq_tanglegram_veaf089 [file supplemental_figure_12_rna3_chaq_tanglegram_veaf089.pdf]
