# Supplementary material for: Constrained reassortment and genotype-specific traits shape the evolutionary landscape of galbut virus
Source: Virus Evol. 2025 Nov 4;11(1):veaf089. doi: 10.1093/ve/veaf089 (PMC12640543; doi:10.1093/ve/veaf089)
Supplement: Supplemental_Figure_Legends_veaf089 [file supplemental_figure_legends_veaf089.docx]

**Supplemental Figure Legends**

**Supplemental Figure 1**: Coverage depth across all coding-complete galbut virus and chaq virus sequences generated in this study, with Genbank accessions indicated. Sample IDs as in **Supp. Table 4**.

**Supplemental Figure 2: Concordance of galbut virus RNA levels measured by RT-qPCR and sequencing.** Scatter plots show concordance between galbut virus RNA levels measured by RT-qPCR (Y axes) and by total RNA sequencing (NGS, X axes). Each point corresponds to a single fly. Galbut virus RNA levels measure by RT-qPCR are normalized to levels of RpL32 mRNA. In (A), the X axis values depict galbut virus RNA1 mapping reads per RpL32 mRNA (NM_079843) mapping reads per kb of their respective lengths. In (B), the X axis values depict galbut virus RNA 1 mapping reads per million host-mapping reads. Regression lines, formulas, and coefficients of determination for linear regressions of log10-transformed values are plotted.

**Supplemental Figure 3: Midpoint rooted maximum likelihood tree of galbut virus RNA 1 coding-complete nucleotide sequences extended data.** Data as shown in Figure 2 but with no clades collapsed. Tip labels for sequences from this study include sample IDs. Sequences recovered from the same sample are connected by lines.

**Supplemental Figure 4:** **Midpoint rooted maximum likelihood tree of galbut virus RNA 2 coding-complete nucleotide sequences extended data.** Sample names are colored by location. Sequences generated by others are indicated with an asterisk (*). Sequences from museum specimens are indicated by a caret (^) (Keene and Stenglein 2024). Support values for select nodes are indicated. Tip labels for sequences from this study include sample IDs. Sequences recovered from the same samples are connected by lines.

**Supplemental Figure 5:** **Midpoint rooted maximum likelihood tree of galbut virus RNA 3 coding-complete nucleotide sequences extended data.** Sample names are colored by location. Sequences generated by others are indicated with an asterisk (*). Sequences from museum specimens are indicated by a caret (^) (Keene and Stenglein 2024).Tip labels for sequences from this study include sample IDs. Sequences recovered from the same samples are connected by lines.

**Supplemental Figure 6:** **Midpoint rooted maximum likelihood tree of chaq virus coding-complete nucleotide sequences extended data.** Data as shown in Figure 3 but with no clades collapsed. Tip labels for sequences from this study include sample IDs. Sequences recovered from the same samples are connected by lines.

**Supplemental Figure 7: Galbut virus RNAs exhibit different ratios of plus (+) strand to minus (-) strand RNA.** Points represent single flies. (A) The fraction of galbut virus-mapping reads derived from +strand RNA for each galbut or chaq virus segment. (B) The number of +strand mapping reads per million host-mapping reads (C) The number of -strand mapping reads per million host-mapping reads. (D) The ratio of +strand mapping reads to -strand mapping reads. The significance levels for adjusted p-values for Wilcoxon rank-sum tests are shown as follows: p < 10^-4^: ****, p < 10^-3^: ***, p < 10^-2^: **, p < 0.05: *. Significance levels for non-significant comparisons are not shown.

**Supplemental Figure 8:** **Galbut virus RNA 3 is more diverse than galbut virus RNAs 1 and 2.** Histograms showing all pairwise nucleotide identities for all sequences for each galbut virus and chaq virus segment.

**Supplemental Figure 9: Galbut virus reassortment involving RNA 1 and RNA 2.** Maximum likelihood midpoint root trees showing all RNA 1 and RNA 2 nucleotide sequences from singly infected samples. Branches with lengths < 0.001 were converted to polytomies. Sequences from the same sample are connected by lines. Selected examples of phylogenetic discordance between pairs of samples consistent with reassortment are highlighted with red lines.

**Supplemental Figure 10:** **Galbut virus reassortment involving RNA 2 and RNA 3.** Maximum likelihood midpoint root trees showing all RNA 2 and RNA 3 nucleotide sequences from singly infected samples. Branches with lengths < 0.001 were converted to polytomies. Sequences from the same sample are connected by lines. Selected examples of phylogenetic discordance of pairs of samples consistent with reassortment are highlighted with red lines.

**Supplemental Figure 11:** **Reassortment involving galbut virus RNA 2 and chaq virus.** Maximum likelihood midpoint root trees showing all RNA 2 and chaq virus nucleotide sequences from samples without evidence of coinfection. Branches with lengths < 0.001 were converted to polytomies. Sequences from the same sample are connected by lines. Selected examples of phylogenetic discordance consistent with reassortment, or a reassortment-like-process, are highlighted with red lines.

**Supplemental Figure 12:** **Reassortment involving galbut virus RNA 3 and chaq virus .** Maximum likelihood midpoint root trees showing all RNA 3 and chaq virus nucleotide sequences from samples without evidence of coinfection. Branches with lengths < 0.001 were converted to polytomies. Sequences from the same sample are connected by lines. Selected examples of phylogenetic discordance consistent with reassortment, or a reassortment-like-process, are highlighted with red lines.

**Supplemental Tables**

**Supplemental Table 1 -** Primer sequences used in this study

**Supplemental Table 2 -** Pairwise Fisher's exact test for differences in galbut virus prevalence at different locations

**Supplemental Table 3 -** Pairwise Fisher's exact test for differences in galbut virus prevalence at different timepoints for longitduinally sampled locations

**Supplemental Table 4 -** Sequenced samples metadata and accessions

**Supplemental Table 5 -** CO1-mapping-based species assignment

**Supplemental Table 6 -** Sites experiencing purifying selection

**Supplemental Table 7** - sites experiencing diversifying selection
