# Supplementary material for: Constrained reassortment and genotype-specific traits shape the evolutionary landscape of galbut virus
Source: Virus Evol. 2025 Nov 4;11(1):veaf089. doi: 10.1093/ve/veaf089 (PMC12640543; doi:10.1093/ve/veaf089)
Supplement: Supplemental_figure_10_RNA2_RNA3_tanglegram_veaf089 [file supplemental_figure_10_rna2_rna3_tanglegram_veaf089.pdf]

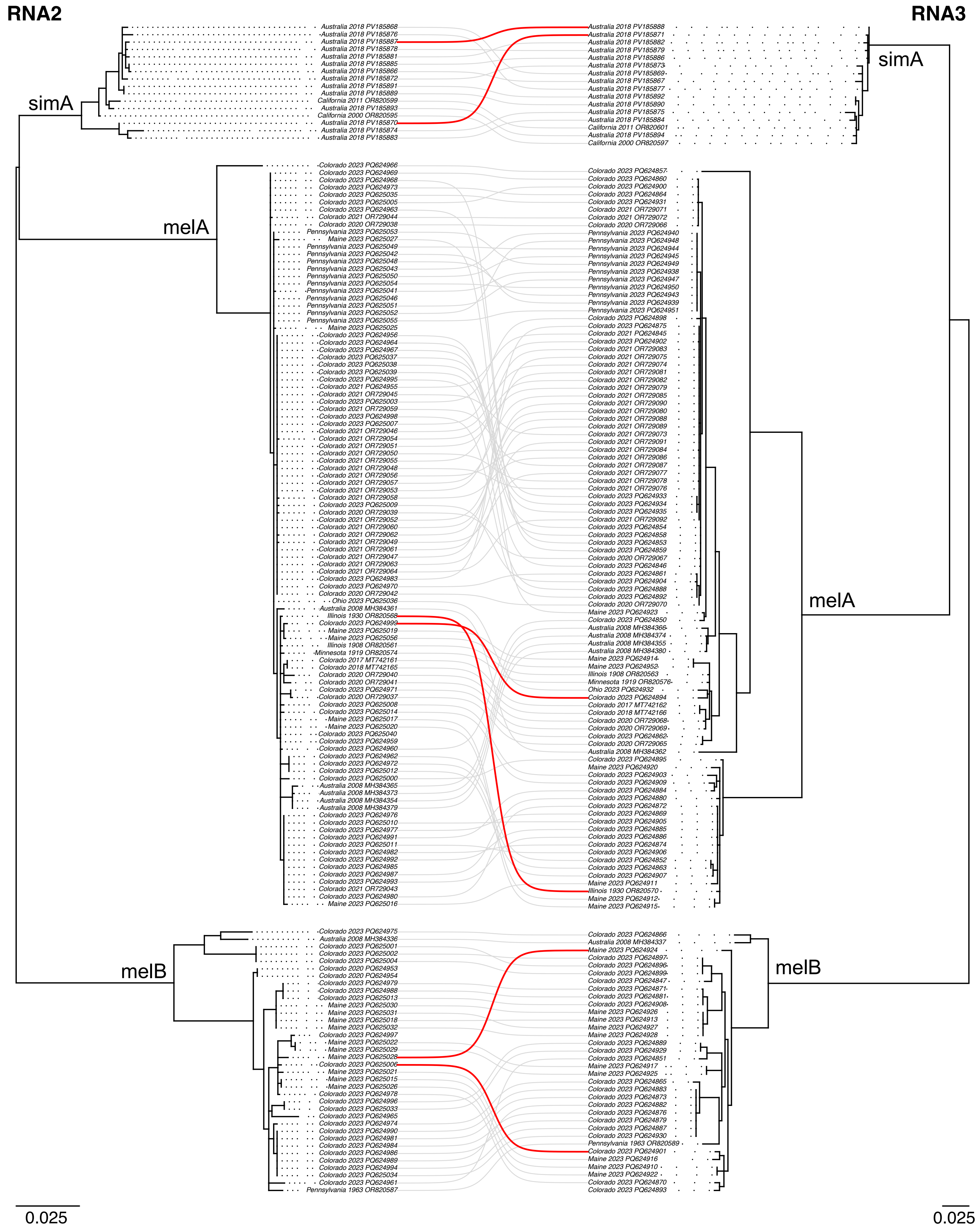
RNA2
RNA3
simA
melA
melB
simA
melA
melB
Australia 2018 PV185868
Australia 2018 PV185876
Australia 2018 PV185887
Australia 2018 PV185878
Australia 2018 PV185881
Australia 2018 PV185885
Australia 2018 PV185866
Australia 2018 PV185872
Australia 2018 PV185891
Australia 2018 PV185889
California 2011 OR820599
Australia 2018 PV185893
California 2000 OR820595
Australia 2018 PV185870
Australia 2018 PV185874
Australia 2018 PV185883
Colorado 2023 PQ624966
Colorado 2023 PQ624969
Colorado 2023 PQ624968
Colorado 2023 PQ624973
Colorado 2023 PQ625035
Colorado 2023 PQ625005
Colorado 2023 PQ624963
Colorado 2021 OR729044
Colorado 2020 OR729038
Pennsylvania 2023 PQ625053
Maine 2023 PQ625027
Pennsylvania 2023 PQ625049
Pennsylvania 2023 PQ625042
Pennsylvania 2023 PQ625048
Pennsylvania 2023 PQ625043
Pennsylvania 2023 PQ625050
Pennsylvania 2023 PQ625054
Pennsylvania 2023 PQ625041
Pennsylvania 2023 PQ625046
Pennsylvania 2023 PQ625051
Pennsylvania 2023 PQ625052
Pennsylvania 2023 PQ625055
Maine 2023 PQ625025
Colorado 2023 PQ624956
Colorado 2023 PQ624964
Colorado 2023 PQ624967
Colorado 2023 PQ625037
Colorado 2023 PQ625038
Colorado 2023 PQ625039
Colorado 2023 PQ624995
Colorado 2021 PQ624955
Colorado 2021 OR729045
Colorado 2023 PQ625003
Colorado 2021 OR729059
Colorado 2023 PQ624998
Colorado 2023 PQ625007
Colorado 2021 OR729046
Colorado 2021 OR729054
Colorado 2021 OR729051
Colorado 2021 OR729050
Colorado 2021 OR729055
Colorado 2021 OR729048
Colorado 2021 OR729056
Colorado 2021 OR729057
Colorado 2021 OR729053
Colorado 2021 OR729058
Colorado 2023 PQ625009
Colorado 2020 OR729039
Colorado 2021 OR729052
Colorado 2021 OR729060
Colorado 2021 OR729062
Colorado 2021 OR729049
Colorado 2021 OR729061
Colorado 2021 OR729047
Colorado 2021 OR729063
Colorado 2021 OR729064
Colorado 2023 PQ624983
Colorado 2023 PQ624970
Colorado 2020 OR729042
Ohio 2023 PQ625036
Australia 2008 MH384361
Illinois 1930 OR820568
Colorado 2023 PQ624999
Maine 2023 PQ625019
Maine 2023 PQ625056
Illinois 1908 OR820561
Minnesota 1919 OR820574
Colorado 2017 MT742161
Colorado 2018 MT742165
Colorado 2020 OR729040
Colorado 2020 OR729041
Colorado 2023 PQ624971
Colorado 2020 OR729037
Colorado 2023 PQ625008
Colorado 2023 PQ625014
Maine 2023 PQ625017
Maine 2023 PQ625020
Colorado 2023 PQ625040
Colorado 2023 PQ624959
Colorado 2023 PQ624960
Colorado 2023 PQ624962
Colorado 2023 PQ624972
Colorado 2023 PQ625012
Colorado 2023 PQ625000
Australia 2008 MH384365
Australia 2008 MH384373
Australia 2008 MH384354
Australia 2008 MH384379
Colorado 2023 PQ624976
Colorado 2023 PQ625010
Colorado 2023 PQ624977
Colorado 2023 PQ624991
Colorado 2023 PQ625011
Colorado 2023 PQ624982
Colorado 2023 PQ624992
Colorado 2023 PQ624985
Colorado 2023 PQ624987
Colorado 2023 PQ624993
Colorado 2021 OR729043
Colorado 2023 PQ624980
Maine 2023 PQ625016
Colorado 2023 PQ624975
Australia 2008 MH384336
Colorado 2023 PQ625001
Colorado 2023 PQ625002
Colorado 2023 PQ625004
Colorado 2020 PQ624953
Colorado 2020 PQ624954
Colorado 2023 PQ624979
Colorado 2023 PQ624988
Colorado 2023 PQ625013
Maine 2023 PQ625030
Maine 2023 PQ625031
Maine 2023 PQ625018
Maine 2023 PQ625032
Colorado 2023 PQ624997
Maine 2023 PQ625022
Maine 2023 PQ625029
Maine 2023 PQ625028
Colorado 2023 PQ625006
Maine 2023 PQ625021
Maine 2023 PQ625015
Maine 2023 PQ625026
Colorado 2023 PQ624978
Colorado 2023 PQ624996
Colorado 2023 PQ625033
Colorado 2023 PQ624965
Colorado 2023 PQ624974
Colorado 2023 PQ624990
Colorado 2023 PQ624981
Colorado 2023 PQ624984
Colorado 2023 PQ624986
Colorado 2023 PQ624989
Colorado 2023 PQ624994
Colorado 2023 PQ625034
Colorado 2023 PQ624961
Pennsylvania 1963 OR820587
Australia 2018 PV185888
Australia 2018 PV185871
Australia 2018 PV185882
Australia 2018 PV185879
Australia 2018 PV185886
Australia 2018 PV185873
Australia 2018 PV185869
Australia 2018 PV185867
Australia 2018 PV185877
Australia 2018 PV185892
Australia 2018 PV185890
Australia 2018 PV185875
Australia 2018 PV185884
California 2011 OR820601
Australia 2018 PV185894
California 2000 OR820597
Colorado 2023 PQ624857
Colorado 2023 PQ624860
Colorado 2023 PQ624900
Colorado 2023 PQ624864
Colorado 2023 PQ624931
Colorado 2021 OR729071
Colorado 2021 OR729072
Colorado 2020 OR729066
Pennsylvania 2023 PQ624940
Pennsylvania 2023 PQ624948
Pennsylvania 2023 PQ624944
Pennsylvania 2023 PQ624945
Pennsylvania 2023 PQ624949
Pennsylvania 2023 PQ624938
Pennsylvania 2023 PQ624947
Pennsylvania 2023 PQ624950
Pennsylvania 2023 PQ624943
Pennsylvania 2023 PQ624939
Pennsylvania 2023 PQ624951
Colorado 2023 PQ624898
Colorado 2023 PQ624875
Colorado 2021 PQ624845
Colorado 2023 PQ624902
Colorado 2021 OR729083
Colorado 2021 OR729075
Colorado 2021 OR729074
Colorado 2021 OR729081
Colorado 2021 OR729082
Colorado 2021 OR729079
Colorado 2021 OR729085
Colorado 2021 OR729090
Colorado 2021 OR729080
Colorado 2021 OR729088
Colorado 2021 OR729089
Colorado 2021 OR729073
Colorado 2021 OR729091
Colorado 2021 OR729084
Colorado 2021 OR729086
Colorado 2021 OR729087
Colorado 2021 OR729077
Colorado 2021 OR729078
Colorado 2021 OR729076
Colorado 2023 PQ624933
Colorado 2023 PQ624934
Colorado 2023 PQ624935
Colorado 2021 OR729092
Colorado 2023 PQ624854
Colorado 2023 PQ624858
Colorado 2023 PQ624853
Colorado 2023 PQ624859
Colorado 2020 OR729067
Colorado 2023 PQ624846
Colorado 2023 PQ624861
Colorado 2023 PQ624904
Colorado 2023 PQ624888
Colorado 2023 PQ624892
Colorado 2020 OR729070
Maine 2023 PQ624923
Colorado 2023 PQ624850
Australia 2008 MH384366
Australia 2008 MH384374
Australia 2008 MH384355
Australia 2008 MH384380
Maine 2023 PQ624914
Maine 2023 PQ624952
Illinois 1908 OR820563
Minnesota 1919 OR820576
Ohio 2023 PQ624932
Colorado 2023 PQ624894
Colorado 2017 MT742162
Colorado 2018 MT742166
Colorado 2020 OR729068
Colorado 2020 OR729069
Colorado 2023 PQ624862
Colorado 2020 OR729065
Australia 2008 MH384362
Colorado 2023 PQ624895
Maine 2023 PQ624920
Colorado 2023 PQ624903
Colorado 2023 PQ624909
Colorado 2023 PQ624884
Colorado 2023 PQ624880
Colorado 2023 PQ624872
Colorado 2023 PQ624869
Colorado 2023 PQ624905
Colorado 2023 PQ624885
Colorado 2023 PQ624886
Colorado 2023 PQ624874
Colorado 2023 PQ624906
Colorado 2023 PQ624852
Colorado 2023 PQ624863
Colorado 2023 PQ624907
Maine 2023 PQ624911
Illinois 1930 OR820570
Maine 2023 PQ624912
Maine 2023 PQ624915
Colorado 2023 PQ624866
Australia 2008 MH384337
Maine 2023 PQ624924
Colorado 2023 PQ624897
Colorado 2023 PQ624896
Colorado 2023 PQ624899
Colorado 2023 PQ624847
Colorado 2023 PQ624871
Colorado 2023 PQ624881
Colorado 2023 PQ624908
Maine 2023 PQ624926
Maine 2023 PQ624913
Maine 2023 PQ624927
Colorado 2023 PQ624928
Colorado 2023 PQ624889
Colorado 2023 PQ624929
Colorado 2023 PQ624851
Maine 2023 PQ624917
Maine 2023 PQ624925
Colorado 2023 PQ624865
Colorado 2023 PQ624883
Colorado 2023 PQ624873
Colorado 2023 PQ624882
Colorado 2023 PQ624876
Colorado 2023 PQ624879
Colorado 2023 PQ624887
Colorado 2023 PQ624930
Pennsylvania 1963 OR820589
Colorado 2023 PQ624901
Maine 2023 PQ624916
Maine 2023 PQ624910
Maine 2023 PQ624922
Colorado 2023 PQ624870
Colorado 2023 PQ624893
0.025
0.025
